# The dose–effect relationship between acupuncture and its effect on primary insomnia: a systematic review and meta-analysis

**DOI:** 10.3389/fpsyt.2025.1501321

**Published:** 2025-02-10

**Authors:** Xiaoni Zhang, Yue Wang, Chengyong Liu, Shan Qin, Liyu Lin, Can Dong, Wenzhong Wu, Zhaoming Chen

**Affiliations:** ^1^ Department of Acupuncture, Nanjing Hospital of Chinese Medicine Affiliated to Nanjing University of Chinese Medicine, Nanjing, Jiangsu, China; ^2^ The First Clinical Medical College, Nanjing University of Chinese Medicine, Nanjing, Jiangsu, China; ^3^ Department of Acupuncture Rehabilitation, Affiliated Hospital of Nanjing University of Chinese Medicine, Nanjing, Jiangsu, China

**Keywords:** acupuncture, dose-effect, primary insomnia, systematic review, meta-analysis

## Abstract

**Background:**

The benefits of acupuncture on primary insomnia (PI) have been well established in previous studies. However, different acupuncture dosages may lead to controversy over its efficacy. Therefore, this systematic review and meta-analysis was conducted to assess the relationship between the dose and efficacy of acupuncture for the treatment of PI.

**Methods:**

Seven databases were searched from inception until May 30, 2024. The included randomized controlled trials (RCTs) with acupuncture for PI on the Pittsburgh Sleep Quality Index (PSQI) scores were divided into three categories according to the therapeutic dose of acupuncture (frequency, session, and course): low dosage, medium dosage, and high dosage. The correlation between the dose and the effect of treatment was analyzed. Risk of bias was assessed using Cochrane Collaboration’s risk of bias tool. Meta-analyses were performed using RevMan v.5.4 and Stata 16.0 software.

**Results:**

A total of 56 studies were included. There were 17 sham acupuncture-controlled RCTs that are notable because of their high quality. Overall, the effect on the reduction of the PSQI scores varied across the different acupuncture dosages. For the frequency of acupuncture, the results showed a significant improvement in the moderate frequency (three sessions per week) and high frequency (five to seven sessions per week) categories. With regard to the acupuncture session, it was shown that moderate session (12–20 sessions) and high session (24–30 sessions) had better effects on the reduction of the PSQI scores, with low session (≤10 sessions) being not significant. For the acupuncture course, there were no differences in the short course (≤2 weeks) and the long course (>4 weeks) between the acupuncture group and the control group. Medium course (3–4 weeks) was considered as the optimal course. In addition, there were no differences between acupuncture and SATV (sham acupuncture therapy at verum points) on the same acupuncture points in the PSQI scores. The results of GRADE assessment demonstrated that the level of evidence was very low to moderate, probably due to the poor methodological quality and the substantial heterogeneity among studies.

**Conclusions:**

A dose–effect relationship was found between the acupuncture dose and the PSQI scores. Although sham acupuncture needling at the same points as those in acupuncture may not be a true placebo control, this was utilized in a minority of studies. Collectively, the data suggest that at least three sessions per week for 3–4 weeks and a total of at least 12 acupuncture sessions would be the optimal clinical response.

**Systematic review registration:**

https://www.crd.york.ac.uk/, identifier CRD42024560078.

## 1 Introduction

Sleep is a vital process, occupying up to a third of the human life span. According to clinical statistics, approximately 25% of people experience unsatisfactory sleep and that 6.0%–10% of individuals meet the diagnostic criteria of insomnia (1). Primary insomnia (PI) is characterized by difficulties in falling asleep and in maintaining sleep and by early morning awakening. It is coupled with daytime consequences such as fatigue, attention deficit, and mood instability. Although insomnia is not a critical disease, long-term insomnia can increase the risk of other physical or mental illnesses or exacerbate existing medical or psychiatric disorders (2, 3). The societal costs of insomnia are substantial. Some studies have shown that poor sleepers cost society approximately 10 times as much as good sleepers (4). Benzodiazepine and non-benzodiazepine hypnotics are recommended for short-term use (maximum 4 weeks), with risks of negative side effects and with limited evidence of their long-term efficacy (5). Although cognitive behavioral therapy (CBT) is the first-line treatment for chronic insomnia, an impediment to its wider utilization is the lack of suitably trained psychologists (6).

Acupuncture has been practiced in China for the prevention and treatment of diseases for thousands of years. Several recently published meta-studies suggested that acupuncture has shown a moderate or large effect in patients with PI when compared with sedative hypnotics alone, control acupuncture (invasive and noninvasive sham controls), and no treatment/waitlist (7, 8). However, these meta-studies have primarily focused on the efficacy of acupuncture and the different acupuncture methods. The adequate acupuncture “dose” with optimal intervention parameters and time table has been overlooked, despite its potential direct impact on trial outcomes. Thus far, there has been no systematic review of the dose–response relationship between acupuncture and its efficacy on PI. Therefore, the purpose of this systematic review was to provide evidence-based recommendations for the dose–response association and the optimal dosage of acupuncture in PI.

## 2 Materials and methods

The systematic review protocol was registered on the International Prospective Register of Systematic Reviews (CRD42024560078). The dose–effect meta-analysis was reported according to the Preferred Reporting Items for Systematic Reviews and Meta-Analyses (PRISMA) statements.

### 2.1 Search strategy

Systematic literature searches of PubMed, The Cochrane Library, Web of Science, EMBASE, China National Knowledge Infrastructure (CNKI), Wan Fang Database, China Biology Medicine (CBM), and the VIP Database were performed from the date of database inception to May 25, 2024, for studies on the effects of acupuncture on PI. The search strategy for PubMed is shown in **Supplementary Table S1**.

### 2.2 Inclusion and exclusion criteria

The inclusion criteria followed the PICOS framework, and only randomized controlled trials (RCTs) in the Chinese and the English language were included: i) PI must be diagnosed according to at least one internationally or nationally recognized diagnostic criterion; ii) acupuncture was limited to manual acupuncture (MA) and electro-acupuncture (EA); iii) studies that compared acupuncture with sham acupuncture or with a sedative hypnotic drug; and iv) the primary outcome was the Pittsburgh Sleep Quality Index (PSQI) score.

The exclusion criteria were as follows: i) clinical trials with fewer than 20 participants in either the intervention or the control group and ii) studies employing non-acupuncture techniques or combined methods in the intervention group, such as a combination of acupuncture with medication, massage, or moxibustion.

### 2.3 Article screening and data extraction

**Figure 1:**
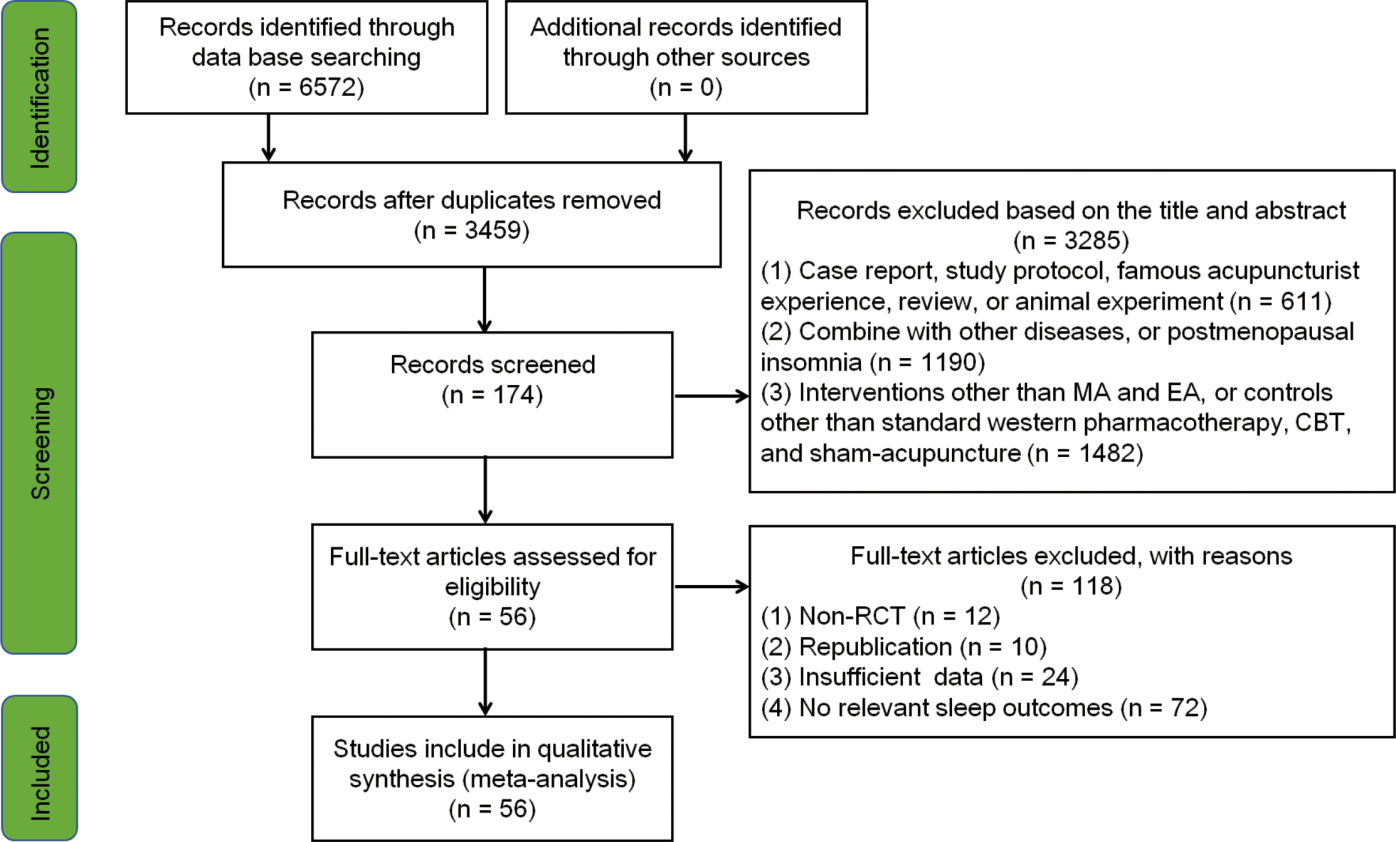
Flowchart of the study search and selection process. *CBT*, cognitive behavioral therapy; *EA*, electro-acupuncture; *MA*, manual acupuncture; *RCT*, randomized controlled trial.

**Figure 1** illustrates the process of study selection. Two researchers (XZ and YW) independently selected the studies and collected the data, importing the identified studies into EndNote X9.0. Where disagreements occurred, a third researcher (CL) was consulted to reach a consensus. The following information was retrieved: study characteristics (i.e., author information, publication year, title, and study design); participant details (i.e., age, gender, duration, and diagnosis); method of intervention/control (i.e., number of treatments and frequency); outcomes [e.g., mean and standard deviation (SD) of the PSQI scores and follow-up].

### 2.4 Risk of bias assessment

Two trained researchers (SQ and LL) independently assessed the risk of bias of the included studies using the Cochrane Collaboration’s risk of bias tool. For any disagreements, a third reviewer (CD) helped to reach consensus. The evaluations included the following categories: random sequence generation, allocation concealment, blinding of participants and personnel, blinding of outcome assessors, completeness of the outcome data, selective reporting, and other bias. For each category, the risk of bias was rated as low, high, or unclear.

### 2.5 Data synthesis and statistical analysis

Data were analyzed using RevMan 5.4 and Stata 16.0 software. For continuous variables, the mean difference (MD) with 95% confidence interval (95%CI) was calculated. For dichotomous variables (effective rate), the relative risk (RR) with 95%CI was determined. The *I* ^2^ statistic was used to assess heterogeneity among the RCTs. An *I* ^2^ >50% indicates heterogeneity, while an *I* ^2^ <50% was assumed to indicate no heterogeneity. In analyses where *p* > 0.1 and *I* ^2^ < 50%, a fixed effects model was applied; otherwise, a random effects model was used. To explore the most suitable or the optimal parameters of acupuncture dose, the included studies were divided into three different groups. For acupuncture frequency, the studies were divided into: low frequency (one to two sessions per week), moderate frequency (three sessions per week), and high frequency (five to seven sessions per week). For acupuncture session, they were classified into: low session (<12 sessions), moderate session (12–20 sessions), and high session (24–30 sessions). According to the course of treatment, the three groups were: short course (≤2 weeks), medium course (3–4 weeks), and long course (>4 weeks). If substantial heterogeneity was detected, subgroup analyses were considered to explore the causes of heterogeneity. Sensitivity analyses were assessed by removing any single study in each group to explore its effect on heterogeneity. Publication bias was evaluated using funnel plots and Egger’s test when at least 10 studies were included.

### 2.6 Quality assessment

To assess the certainty of evidence, the GRADEpro online tool (http://gdt.gradepro.org/app#projects) was used to perform the evaluation and followed the recommended procedures for grading (high, moderate, low, or very low).

## 3 Results

A total of 6,572 articles were initially retrieved from the searches. After removing the duplicates and further screening, 56 RCTs (involving 4,019 participants) were ultimately included in the meta-analyses. The literature screening process is summarized in **Figure 1**.

### 3.1 Study characteristics

The features of the included studies are shown in **Table 1**. Of the included studies, 55 (9–22, 24–64) were conducted in China and one (23) was conducted in Korea. Of those conducted in China, four were published in English (22–25) and 52 in Chinese (9–21, 26–64). There were 17 RCTs (9–25) that compared acupuncture with sham acupuncture, while 39 RCTs (21–59) compared acupuncture with Western medication (sedative hypnotics).

**Table 1:** Characteristics of the 56 studies included in the analyses.

Study (first name, year)	Cases (T/C)/n	Age(T/C)/year	Sex(T/C)/n of man	Insomnia duration (months = m, years = y)	Diagnostic system	Treatment group				Control group	Outcome measure	Follow-up times
						Type	Main acupuncture points	Session	Curse (week)			
Mo 2016 (9)	T/n = 30 C/n = 30	T/49.40 ± 2.931 C/49.20 ± 3.078	T/n = 5 C/n = 6	T/1.3 ± 0.2 y C/1.1 ± 0.3 y	DSM-5	EA; -5 times/week	GV20, HT7, SP6	25	5	Sham acupuncture (SATS)	PSQI	The 4th week
Li 2017 (10)	T/n = 30 C/n = 27	T/47.00 ± 0.50 C/48.00 ± 1.00	T/n = 3 C/n = 6	T/105.73 ± 95.24 m C/104.96 ± 86.00 m	CCMD-3	MA; -3 times/week	GV20, GV24, EX-HN1, GB 13, HT7, PC6, SP6	12	4	Sham acupuncture (SATS)	PSQI	The 4th week, the 8th week
Dong 2017 (11)	T/n = 32 C/n = 31	T/45 ± 18 C/44 ± 21	T/n = 9 C/n = 11	T/1.6 ± 1. 4 y C/1.7 ± 1.3 y	ICSD-3	MA; -3 times/week	GV29, GV20, GV14	12	4	Sham acupuncture (SATS)	PSQI	the 4th week
Zhu 2018 (12)	T/n = 30 C/n = 32	T/48.97 ± 6.29 C/48.56 ± 6.07	T/n = 14 C/n = 13	NA	DSM-5	EA; -5 times/week	EX-HN1, HT7, SP6, LR3	20	4	Sham acupuncture (SATS)	PSQI	The 4th week
Guo 2017 (13)	T/n = 30 C/n = 29	T/47.75 ± 3.71 C/48.31 ± 3.14	T/n = 3 C/n = 7	T/8.31 ± 2.40 m C/7.75 ± 1.60 m	DSM-4	MA; -3 times/week	GV20, GV24, EX-HN1, GB13, HT7, PC6, SP6	12	4	Sham acupuncture (SATS)	PSQI	The 8th week
Huang 2019 (14)	T/n = 38 C/n = 38	T/41.13 ± 10.51 C/39.89 ± 9.06	T/n = 23 C/n = 22	NA	ICSD-3	MA; -3 times/week	BL18, BL20, LR3, SP3, LR2, SP2	9	3	Sham acupuncture (SATS)	PSQI	NO follow-up
Wan 2018 (15)	T/n = 29 C/n = 28	T/45.38 ± 13.03 C/44.32 ± 12.58	T/n = 10 C/n = 11	T/2.67 ± 3.16 m C/2.24 ± 3.69 m	CCMD-3	MA; -3 times/week	GV20, GV24, GV29, HT7, SP6	12	4	Sham acupuncture (SATV)	PSQI	NO follow-up
Yuan 2018 (16)	T/n = 40 C/n = 42	T/48.13 ± 4.31 C/47.41 ± 5.84	T/n = 33 C/n = 36	T/4.81 ± 1.24 y C/4.26 ± 1.13 y	DSM-4	MA; -3 times/week	GV20, GV24, EX-HN1, EX-HN22, GB13, HT7, PC6, SP 6	12	4	Sham acupuncture (SATS)	PSQI	NO follow-up
Zhang 2019 (17)	T/n = 51 C/n = 53	T/47.29 ± 11.84 C/51.13 ± 12.56	T/n = 11 C/n = 10	T/6.55 ± 6.47y C/6.48 ± 7.35y	ICSD-3	MA; -every other day	GV20, HT7, SP6, KI6, BL62, EX-HN22, BL15, BL20, ST36	14	4	Sham acupuncture (SATS)	PSQI	NO follow-up
Xi 2021 (18)	T/n = 29 C/n = 29	T/44 ± 12 C/41 ± 12	T/n = 12 C/n = 13	T/6.6 ± 4.4 y C/5.7 ± 4.7y	ICSD-3	EA; -3 times/week	GV20, GV24, GV29, EX-HN22, HT7, SP6	12	4	Sham acupuncture (SATS)	PSQI	NO follow-up
Huo 2023 (19)	T/n = 28 C/n = 27	T/48 ± 9 C/46 ± 8	T/n = 14 C/n = 16	T/21~5 m C/12~48 m	DSM-IV	MA; -3 times/week	GV20, GV24, GB13, HT7, PC6, SP6	12	4	Sham acupuncture (SATS)	PSQI	The 4th week
Wu W.Z. 2021 (20)	T/n = 34 C/n = 32	T/46.22 ± 11.91 C/47.00 ± 13.59	T/n = 15 C/n = 11	T/2.80 ± 3.32 y C/3.10 ± 4.23 y	ICSD-3	EA; -3 times/week	GV20, GV29, HT7, SP6, BL13, BL15, BL18, BL20, BL23	12	4	Sham acupuncture (SATS)	PSQI	The 4th week
Jiang 2022 (21)	T/n = 24 C/n = 26	T/3.67 ± 14.08 C/37.18 ± 1.8	T/n = 9 C/n = 11	NA	DSM-5	MA; -3 times/week	GV20, GV24, EX-HN1, GB13, HT7, PC6, SP6	12	4	Sham acupuncture (SATS)	PSQI	The 3th month
Yang 2009 (22)	T/n = 29 C/n = 28	T/48.3 ± 9.5 C/47.8 ± 8.6	T/n = 8 C/n = 6	T/11.23 ± 5.57 m C/10.20 ± 5.22 m	DSM-IV	EA; -3 times/week	EX-HN3, GV20, EX-HN1, EX-HN22	12	4	Sham acupuncture (SATV)	PSQI	NO follow-up
Lee 2020 (23)	T/n = 40 C/n = 43	T/51.78 ± 2.54 C/52 ± 2.48	T/n = 9 C/n = 9	T/76.37 ± 22.83 m C/72.35 ± 18.76 m	DSM-IV	EA; -5 times/week	GV20, EX-HN3, HT7, PC6, BL63, KI4	10	2	Sham acupuncture (SATS)	PSQI	The 4th week, the 8th week
Liu 2021 (24)	T/n = 31 C/n = 29	T/47.17 ± 14.08 C/45.59 ± 12.65	T/n = 13 C/n = 10	T/2.71 ± 3.22 m C/2.90 ± 3.55 m	ICSD-3	EA; -5 times/week	GV20, GV29, HT7, SP6	12	4	Sham acupuncture (SATS)	PSQI	The 3th month
Zhang 2023 (25)	T/n = 43 C/n = 45	T/38.09 ± 13.33 C/39.41 ± 13.93	T/n = 15 C/n = 14	T/3.2 ± 1.5 y C/4.2 ± 1.1 y	DSM-V	MA; -5 times/week	EX-HN22, PC6, HT7, LI4, ST36, KI6, BL62, LR3	10	2	Sham acupuncture (SATV)	PSQI	The 4th week, the 16th week, the 40th week
Wu 2007 (26)	T/n = 32 C/n = 30	T/54.8750 ± 1.8249 C/53.5000 ± 1.7621	T/n = 15 C/n = 15	T/73.2188 ± 2.0154 m C/75.4333 ± 2.9780 m	CCMD-3	MA; -5 times/week	GV20, EX-HN1, GV24, GB13, HT7	20	4	Estazolam; -1 mg/day for 4 weeks	PSQI	NO follow-up
Bai 2011 (27)	T/n = 30 C/n = 30	T/38.36 ± 13.52 C/39.23 ± 13.14	T/n = 13 C/n = 14	T/46.89 ± 16.32 d C/43.35 ± 15.94 d	CCMD-3	EA; -5 times/week	BL62, KI6, BL59, BL61, KI8, KI2, BL1	30	4	Estazolam; -1 mg/day for 1 month	PSQI	NO follow-up
Meng 2011 (28)	T/n = 30 C/n = 30	18~60	T/n = 16 C/n = 17	2 m, 16 y	CCMD-3	MA; -7 times/week	BL15, BL18, CV14, LR14	28	4	Estazolam; -2 mg/day for 4 weeks	PSQI	NO follow-up
Luan 2012 (29)	T/n = 30 C/n = 30	21~60	T/n = 7 C/n = 9	T/27.77 ± 23.84 C/30.03 ± 24.35	CCMD-3	EA; -6 times/week	GB20, gongxue	24	4	Estazolam; -1 mg/day for 4 weeks	PSQI	NO follow-up
Xia 2012 (30)	T/n = 42 C/n = 40	T/18~63 C/19~65	T/n = 18 C/n = 17	T/2 m, 4 y C/2 m, 5 y	CCMD-3	MA; -5 times/week	GV26, GV24, GV20, GV17, GV16, GV14, GV11, GV10, GV9, GV4, CV6, CV12, CV14, CV15, CV17, CV24, GV29, BL15, BL23	20	4	Estazolam; -1~2 mg/day for 4 weeks	PSQI	NO follow-up
Liao 2013 (31)	T/n = 30 C/n = 30	T/45.23 ± 14.10 C/43.10 ± 12.16	T/n = 7 C/n = 9	T/14.27 ± 7.78 d C/16.47 ± 7.49 d	CCMD-2	EA; -1 time/day	EX-HN22, GB 20	20	3	Estazolam; -1 mg/day for 3 weeks	PSQI	NO follow-up
Li 2013 (32)	T/n = 45 C/n = 45	T/36.74 ± 9.31 C/35.66 ± 8.99	T/n = 23 C/n = 21	T/25.3 ± 7.6 m C/25.9 ± 10.3 m	CCMD	EA; -1 time/day	EX-HN5, EX-HN1, ST36, SP6	24	4	Diazepam; -5 mg/day for 10 days	PSQI	NO follow-up
Li 2014 (33)	T/n = 30 C/n = 30	T/29.43 ± 53.3 C/32.00 ± 6.43	T/n = 10 C/n = 13	T/11.23 ± 5.57 m C/10.20 ± 5.22 m	CCMD-3	EA; -1 time/day	EX-B2 (T5-L2), EX-HN1, EX-HN22	30	32 d	Estazolam -1 mg/day for 1month	PSQI	NO follow-up
Liu Y.Y. 2014 (34)	T/n = 35 C/n = 35	T/43.56 ± 13.16 C/41.36 ± 12.50	T/n = 15 C/n = 14	No reported	CCMD-2	MA; -6 times/week	Sishenzhen, Dingshenzhen	12	2	Estazolam; -1 mg/day for 2 weeks	PSQI	NO follow-up
Liu Y. 2014 (35)	T/n = 30 C/n = 30	T/46.31 ± 18.26 C/41.29 ± 17.64	T/n = 17 C/n = 12	T/3.78 ± 1.98 m C/3.54 ± 1.52 m	CCMD-3	MA; -6 times/week	GV24, GV20, GV18, GV17, GV16, GV14, GV11	24	4	Estazolam; -1 mg/day for 4 weeks	PSQI	NO follow-up
Wang 2014 (36)	T/n = 40 C/n = 40	T/51.4 ± 2.3 C/53.1 ± 2.1	T/n = 15 C/n = 14	T/3.2 ± 1.5 y C/4.2 ± 1.1 y	CCMD-2	MA; -1 time/day	GB20, SP6, HT7, PC4	30	30 d	Estazolam; -1~2 mg/day for 1 month	PSQI	NO follow-up
Wu 2014 (37)	T/n = 31 C/n = 29	T/19~63 C/18~65	T/n = 12 C/n = 11	T/2 m ~ 8 y C/3 m ~ 10 y	CCMD-3	MA; -6 times/week	PC6, HT7, LR3, GB34, EX-HN1	10	2	Estazolam; -1 mg/day for 10 days	PSQI	NO follow-up
Zhu 2014 (38)	T/n = 30 C/n = 30	T/41.13 ± 10.13 C/40.90 ± 10.39	T/n = 13 C/n = 16	T/16.93 ± 5.24 m C/17.62 ± 5.17 m	DSM-IV	EA; -7 times/week	BL13, BL15, BL18, BL20, BL23	28	4	Estazolam; -1 mg/day for 4 weeks	PSQI	NO follow-up
Liu 2015 (39)	T/n = 96 C/n = 95	T/21~70 C/23~68	No reported	T/3 m ~ 32 m C/4 m ~ 34 m	CCMD-3	MA; -1 time/day	EX-HN1, EX-HN22, SP6, HT7, KI3	20	24 d	Estazolam; -1 mg/day for 4 weeks	PSQI	NO follow-up
Wang Y.Y. 2015 (40)	T/n = 30 C/n = 30	T/44.67 ± 8.36 C/45.13 ± 9.82	T/n = 13 C/n = 12	T/9.3 ± 4.80 C/9.5 ± 4.41	CCMD-3	MA; -6 times/week	BL13, BL15, BL18, BL20, BL23, GV20, GV29, ST25, CV4, CV6, PC6, HT7, BL62, KI6, SP6	24	4	Estazola; -1 mg/day for 4 weeks	PSQI	NO follow-up
Zhu 2015 (41)	T/n = 30 C/n = 30	T/37 ± 4.5 C/35 ± 6.7	T/n = 7 C/n = 8	T/22.65 ± 23.80 C/30.21 ± 24.43	CCMD-3	EA; -1 time/day	GV20, EX-HN1, EX-HN5, GB20, HT7, SP6, KI6	21	3	Estazolam; -1 mg/day for 3 weeks	PSQI	NO follow-up
Jia 2016 (42)	T/n = 40 C/n = 40	T/55.5 ± 3.1 C/55.7 ± 3.2	T/n = 23 C/n = 24	No reported	CCMD-3	MA; -1 time/day	EX-HN1, GV20	14	2	Diazepam; -5 mg/day for 2 weeks	PSQI	NO follow-up
Pei 2016 (43)	T/n = 30 C/n = 30	T/48.86 ± 9.05 C/53.30 ± 6.05	T/n = 12 C/n = 19	T/8.50 ± 2.71 m C/7.80 ± 2.44 m	CCMD-3	EA; -1 time/day	Six acupionts (the acupoints are not fixed every time)	20	20 d	Estazolam; -1 mg/day for 20 days	PSQI	NO follow-up
Wang 2016 (44)	T/n = 34 C/n = 34	T/53 ± 13.43 C/53 ± 11.37	T/n = 9 C/n = 11	T/2.35 ± 2.02 y C/2.07 ± 1.10 y	CCMD-3	MA; -5 times/week	GV20, GV24, EX-HN1, EX-HN14, GB20, HT7, LR3, KI3, CV12, ST25, SP9	20	4	Estazolam; -1 mg/day for 4 weeks	PSQI	NO follow-up
Lin 2016 (45)	T/n=29 C/n=30	T/32.83 ± 3.54 C/33.02 ± 4.08	T/n=13 C/n=12	T/5.28 ± 1.14m C/5.51 ± 0.98m	CCMD-3	EA; -1 time/day	HT7, KI3, HT5, KI4, GV20	20	20 d	Diazepam; -5 mg/day for 20 days	PSQI	NO follow-up
Hu 2017 (46)	T/n = 50 C/n = 50	T/50.28 ± 8.91 C/49.03 ± 10.27	T/n = 27 C/n = 22	T/0.73 ± 0.17 y C/0.75 ± 0.09 y	CCMD-3	MA; -6 times/week	EX-HN1, GV 20, GV24, EX-HN22, HT 7, PC6	24	28 d	Alprazolam; -0.4 mg/day for 4 weeks	PSQI	NO follow-up
Liu 2017 (47)	T/n = 31 C/n = 30	T/47 ± 10 C/47 ± 10	T/n = 10 C/n = 10	T/14.0 ± 6.5 C/14.0 ± 6.3	CCMD-3	MA; -5 times/week	EX-HN1, EX-HN22, HT7, SP6, KI6, BL62	20	4	Estazolam; -1~2 mg/day for 4 weeks	PSQI	NO follow-up
Shao 2017 (48)	T/n = 56 C/n = 56	T/44.6 ± 13.5 C/45.8 ± 14.1	T/n = 20 C/n = 22	T/6.3 ± 2.4 m C/6.2 ± 2.5	CCMD-3	MA; -1 time/day	GV20, EX-HN1, EX-HN22, EX-HN14, GB20	30	30 d	Zopiclone; -7.5 mg/day for 1 month	PSQI	NO follow-up
Ren 2017 (49)	T/n = 30 C/n = 29	T/56.8 ± 9.14 C/56.2 ± 8.69	T/n = 11 C/n = 11	T/52.1 ± 11.2 C/54.5 ± 12.1	CCMD-3	MA; -5 times/week	BL20, BL15, SP6, ST36, HT7, PC6	20	4	Eszopiclone; -1~3 mg/day for 4 weeks	PSQI	NO follow-up
Zhou 2018 (50)	T/n = 30 C/n = 30	T/49.1 ± 16.7 C/48.7 ± 15.4	T/n = 12 C/n = 14	T/14.0 ± 10.5 C/14.1 ± 10.4	CCMD-3	MA; -7 times/week	GV23, GV29, GV20, EX-HN1, PC6, HT7, SP6, KI3	28	4	Estazolam; -1 mg/day for 4 weeks	PSQI	NO follow-up
Sun 2018 (51)	T/n = 46 C/n = 46	No reported	T/n = 23 C/n = 25	T/2.1 ± 0.9y C/2.3 ± 0.7y	CCMD-3	MA; -5 times/week	GV26, GV24, GV20, GV17, GV11, GV10, CV6, CV17, GV29, BL15, BL23	20	4	Estazolam; -1 mg/day for 4 weeks	PSQI	NO follow-up
Zhong 2019 (52)	T/n = 44 C/n = 44	T/44.3 ± 3.9 C/45.3 ± 3.2	T/n = 18 C/n = 20	T/5.9 ± 3.3 y C/5.8 ± 3.1 y	CCMD-3	MA; -1 time/day	SP6, EX-HN1, PC6, HT7	28	4	Alprazolam;-0.5~0.8 mg/day for 4 weeks	PSQI	NO follow-up
Zhu 2019 (53)	T/n = 30 C/n = 30	T/42.08 ± 13.11 C/44.51 ± 11.47	T/n = 16 C/n = 12	T/4.71 ± 0.30 C/4.56 ± 0.78	CCMD-3	MA; -5 times/week	GV20, GV29, EX-HN5, GV26, CV24, PC6, HT7, HT8, CV17, ST36, SP6, KI3, SP4	20	4	Estazolam; -1 mg/day for 4 weeks	PSQI	NO follow-up
Chen 2020 (54)	T/n = 43 C/n = 42	T/52.33 ± 10.00 C/52.43 ± 9.67	T/n = 20 C/n = 19	1 m ~ 11 y	CCMD-3	MA; -1 time/day	CV14, ST25, CV6, CV4, GV24, HT7	20	3	Estazolam; -1~2 mg/day for 3 weeks	PSQI	The 5th week follow- up
Dong 2020 (55)	T/n = 30 C/n = 30	T/63.6 ± 23.1 C/65.2 ± 4.0	T/n = 15 C/n = 14	T/20.2 ± 7.4 C/20.4 ± 6.0	CCMD-3	MA; -3 times/week	GV20, GV16, GB15, GB8, GB20, LR3, GB41, GB43, ST36, BL7, BL8, BL9, GB17, GB18	12	4	Estazolam; -2 mg/day for 4 weeks	PSQI	NO follow-up
Li 2020 (56)	T/n = 30 C/n = 30	T/43.4 ± 10.02 C/43.03 ± 8.86	T/n = 14 C/n = 13	T/11.6 ± 4.91m C/12.16 ± 4.58 m	CCMD-3	MA; -5 times/week	EX-HN1, GV24, GV29, PC6, SP6, BL10, EX-HN15, BL11	20	4	Estazolam; -1 mg/day for 4 weeks	PSQI	NO follow-up
Xu 2020 (57)	T/n = 30 C/n = 30	T/48.3 ± 7.8 C/47.4 ± 8.1	T/n = 14 C/n = 9	T/30.35 ± 18.22 d C/32.29 ± 19.18 d	CCMD-3	MA; -5 times/week	LI 20, EX-HN22, ST36, HT7, PC6, SP6	12	4	Estazolam; -2 mg/day for 4 weeks	PSQI	NO follow-up
Fan 2021 (58)	T/n = 50 C/n = 50	T/44.38 ± 8.57 C/43.88 ± 7.91	T/n = 22 C/n = 23	T/6.14 ± 2.47 C/6.26 ± 2.44	CCMD-3	MA; -6 times/week	CV12, ST25, CV4, HT7, ST36, PC6, PC4, LR3, PC7, KI3, SP3	18	3	Alprazolam; -0.4mg/day for 4 weeks	PSQI	NO follow-up
Wu 2021 (59)	T/n = 30 C/n = 29	T/41 ± 10 C/42 ± 10	T/n = 12 C/n = 10	T/13.4 ± 5.3 C/14.2 ± 4.8	ICSD-3	MA; -3 times/week	GV20, GV24, GV29, HT7, SP6	12	4	Estazolam; -1 mg/day for 4 weeks	PSQI	NO follow-up
Huang 2021 (60)	T/n = 50 C/n = 49	T/49.74 ± 10.94 C/46.63 ± 12.77	T/n = 14 C/n = 14	T/3.71 ± 3.58 C/3.72 ± 4.36	ICSD-3	MA; -1 time/day	LI4, LR3, GB20, GV20, HT7, SP6	28	4	Eszopiclone; -3 mg/day for 4 weeks	PSQI	The 4th week
Zhang 2022 (61)	T/n = 30 C/n = 30	T/47.05 ± 12.10 C/48.02 ± 13.51	T/n = 11 C/n = 13	T/26.7 ± 6.3 C/27.3 ± 6.9	ICSD-3	MA; -6 times/week	CV12, CV10, CV6, CV4, ST 24, ST26, KI17, KI13, SP6, LR2, PC7	12	2	Estazolam; -1~2 mg/day for 4 weeks	PSQI	NO follow-up
Wang 2022 (62)	T/n = 30 C/n = 30	T/40.80 ± 12.97 C/41.43 ± 9.09	T/n = 3 C/n = 4	T/31.40 ± 5.90 m C/30.80 ± 5.60	ICSD-3	MA; -5 times/week	GV29, GV20, GV24, PC6, ST36, ST25, CV6, CV13, CV12, CV10	30	6	Eszopiclone; -2 mg/day for 6 weeks	PSQI	NO follow-up
Wu 2023 (63)	T/n = 45 C/n = 45	T/43 ± 8 C/43 ± 8	T/n = 22 C/n = 24	T/14.1 ± 5.6 m C/14.1 ± 5.4 m	ICSD-3	MA; -6 times/week	EX-HN1, HT7, SP6	24	4	Estazolam; -1 mg/day for 4 weeks	PSQI	NO follow-up
Liu 2024 (64)	T/n = 49 C/n = 48	T/46 ± 11 C/47 ± 10	T/n = 17 C/n = 16	T/13.9 ± 5.8 C/14.3 ± 5.9	ICSD-3	MA -5 times/week	GV20, HT7, SP6, BL62, KI6, BL15, BL23	20	4	Lorazepam; -0.5 ~ 1 mg/day for 4 weeks	PSQI	NO follow-up

C, control; CCMD-3, Chinese Classification of Mental Disorders version 3; DSM-IV, Diagnostic and statistical manual of mental disorders 4th edition; DSM-V, Diagnostic and statistical manual of mental disorders 5th edition; EA, electroacupuncture; ICSD-2, International Classification of Sleep Disorders version 2; ICSD-3, International Classification of Sleep Disorders version 3; MA, manual acupuncture; NR, not reported; PSQI, Pittsburgh Sleep Quality Index; SATS, sham acupuncture needling at different points compared with the acupuncture group; SATV, sham acupuncture needling at the same acupuncture points as the acupuncture group.

### 3.2 Risk of bias assessment

**Figure 2:**
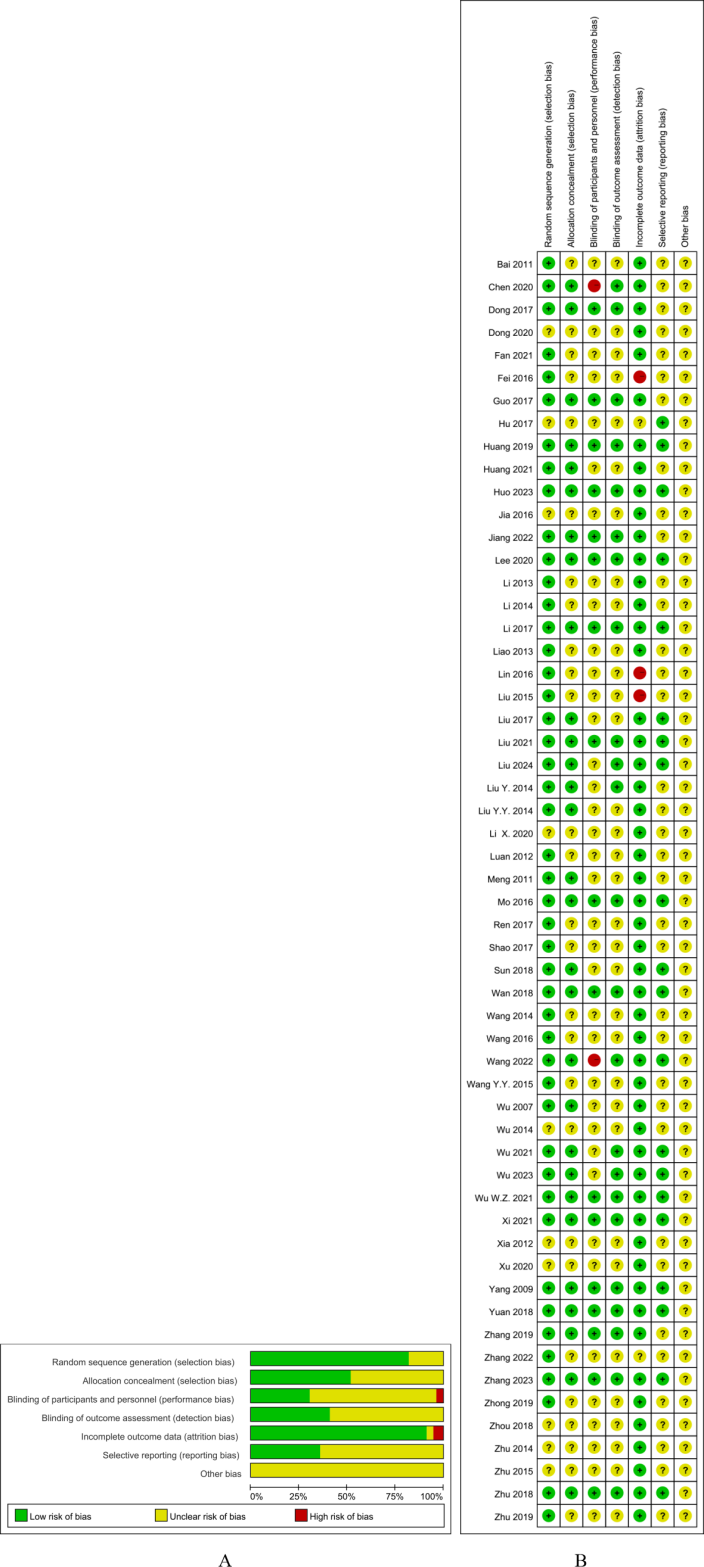
**(A)** Risk of overall bias. **(B)** Risk of bias in individual studies.

The parameters of quality assessment included the following:

- Random sequence generation: Of the 56 RCTs, 46 (9–29, 31–36, 39, 40, 43–45, 47–53, 58–64) (82.1%) had a low risk of bias for random sequence generation.
- Allocation concealment: There were 29 RCTs (9–22, 28, 34, 35, 47, 51, 54, 56, 59, 60, 62–64) that were judged to have a low risk of bias for this item.
- Blinding of participants and personnel: As acupuncture must be performed by a qualified professional, it was impossible to blind the acupuncturists. Only 17 (30.4%) of the RCTs (9–24) that used sham acupuncture as a control involved blinding of the patients.
- Blinding of outcome assessment: There were 23 RCTs (9–25, 35, 54, 59, 62–64) (41.1%) that involved blinding of the data evaluators.
- Incomplete outcome data: A total of 51 RCTs (9–38, 40–42, 44, 47–60, 62–64) (91.1%) were rated as showing a low risk of bias for incomplete outcome data.
- Selective reporting: Of the RCTs, 20 (35.7%) (9, 10, 12, 14–16, 18–20, 22–25, 46, 47, 50, 59, 62–64) showed a low risk of bias as their protocols were registered.
- None of the RCTs described other sources of bias and were rated as showing unclear risk (**Figure 2**).

## 4 Meta-analysis

The 56 RCTs could be classified into two groups based on the comparator used: 1) acupuncture *vs*. sham acupuncture (17 RCTs) and 2) acupuncture *vs*. Western medication (sedative hypnotics; 39 RCTs).

### 4.1 Acupuncture *vs*. sham acupuncture

**Figure 3:**
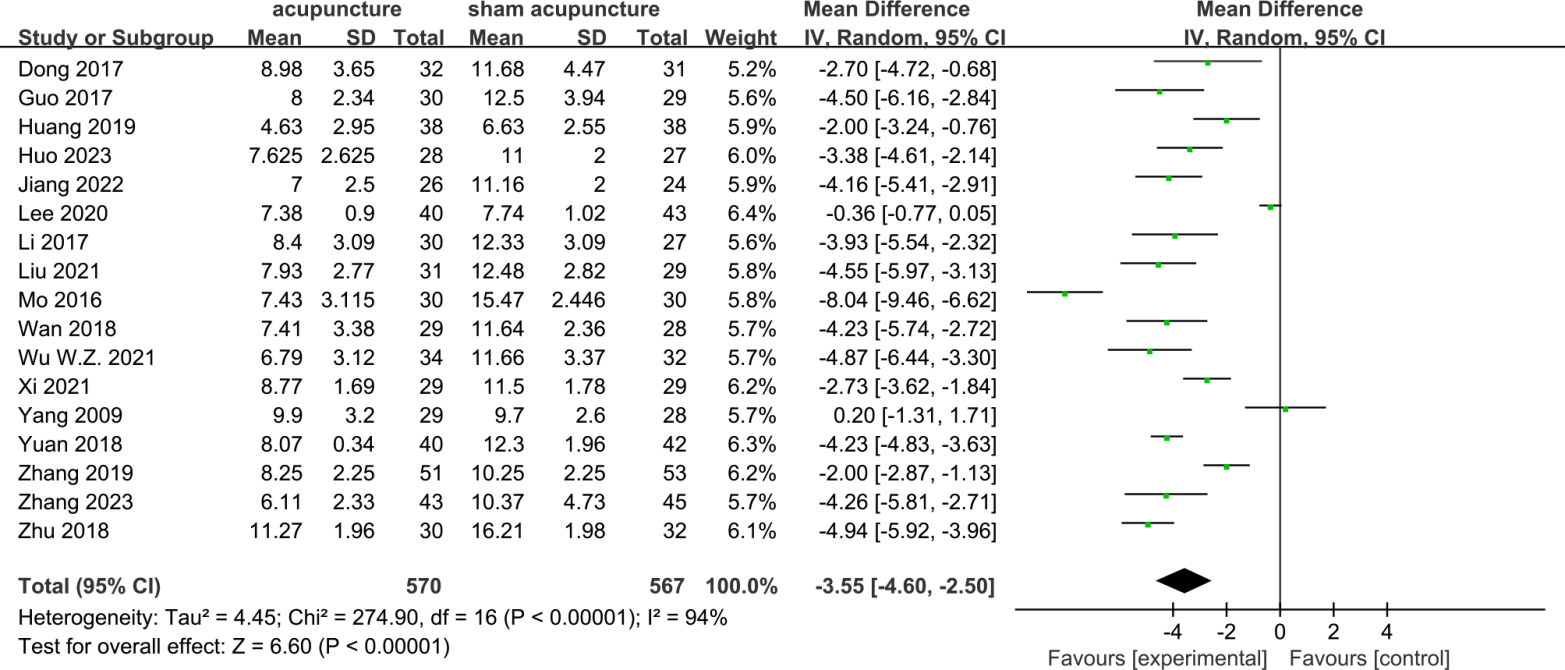
Forest plots of acupuncture *vs*. sham acupuncture for the Pittsburgh Sleep Quality Index (PSQI) after treatment.

**Figure 4:**
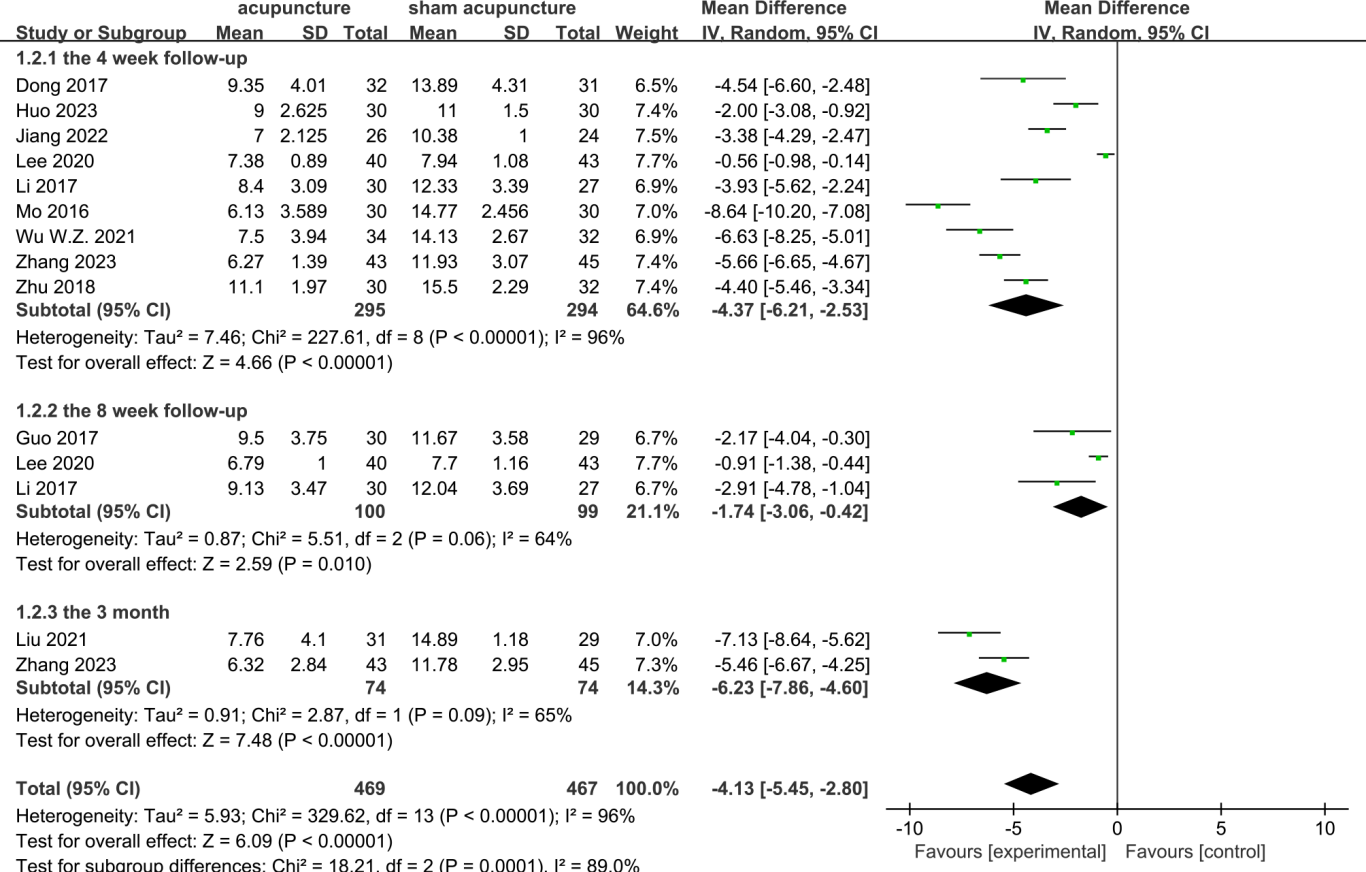
Forest plots of acupuncture *vs*. sham acupuncture for the Pittsburgh Sleep Quality Index (PSQI) at the 4-week, 8-week, and 3-month follow-up.

PSQI scores were reported in all 17 RCTs (9–25). The results of the meta-analysis revealed that acupuncture significantly lowered the PSQI scores compared with sham acupuncture (MD = −3.55, 95%CI = −4.6 to −2.5, *p* < 0.00001, *I* ^2^ = 94%) (**Figure 3**). Due to the high heterogeneity, a random effects model was used. At the 4-week follow-up, eight RCTs (9–12, 19, 20, 23, 25) showed that acupuncture was associated with lowering of the PSQI scores compared with sham acupuncture (MD = −4.37, 95%CI = −6.21 to −2.53, *p* < 0.00001, *I* ^2^ = 96%). At the 8-week follow-up, three RCTs (18, 65, 66) reported that acupuncture resulted in a significant reduction of the PSQI scores compared with sham acupuncture (MD = −1.74, 95%CI = −3.06 to −0.42, *p* = 0.01, *I* ^2^ = 64%). At the 3-month follow-up, two RCTs (16, 19) showed acupuncture to have a significant difference compared with sham acupuncture (MD = −6.23, 95%CI = −7.86 to −4.60, *p* < 0.00001, *I* ^2^ = 65%) (**Figure 4**). Subgroup analyses were performed to determine the heterogeneity of the outcomes. For the different acupuncture methods (MA or EA) (**Supplementary Figure S1**) and the different number of acupoints (≥10 or <10) (**Supplementary Figure S2**), the findings suggested that acupuncture was significantly more effective than sham acupuncture in both subgroups. However, there were no statistically significant associations with the therapeutic effect (MA *vs*. EA: *Chi* ^2^ = 0.00, *df* = 1, *p* = 0.94; ≥10 *vs*. <10: *Chi* ^2^ = 0.24, *df* = 1, *p* = 0.64). The results of the sensitivity analysis showed that it remained essentially unchanged after omitting any one study, suggesting that the research results were credible and relatively stable (**Supplementary Figure S3**). The results of Egger’s test (*p* = 0.009) suggested a potential risk of publication bias, as indicated by the asymmetry in the funnel plots (**Supplementary Figure S4**).

### 4.2 Sham acupuncture type

**Figure 5:**
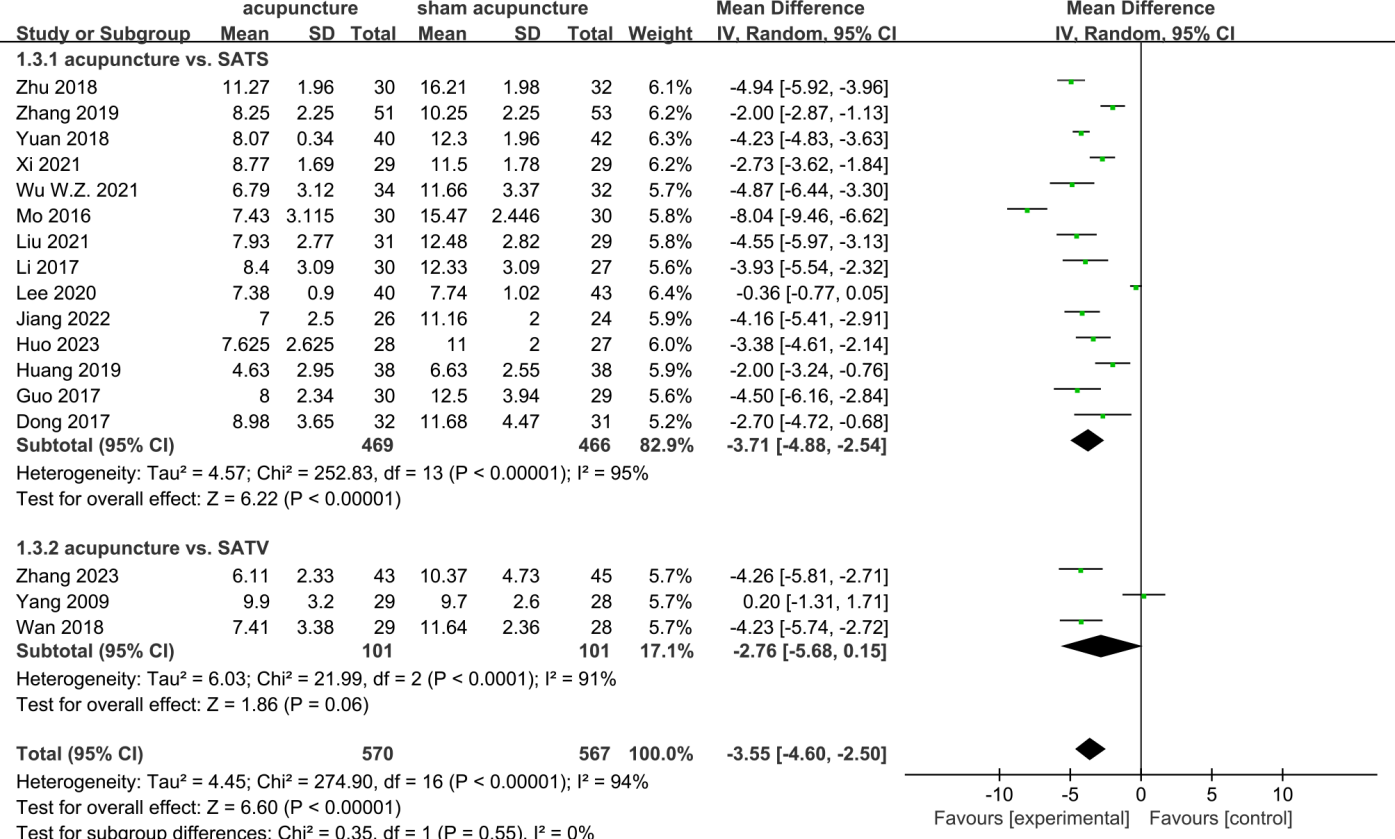
Forest plots of the different sham acupuncture types (SATS or SATV) for the Pittsburgh Sleep Quality Index (PSQI).

In order to evaluate the specific effects of acupuncture, sham acupuncture was classified according to the needling points as follows: 1) SATS (sham acupuncture therapy), which is sham acupuncture needling at different points compared with the acupuncture group, and 2) SATV (sham acupuncture therapy, verum), which is sham acupuncture needling at the same acupuncture points as the acupuncture group. Of the RCTs, 14 (9–14, 16–21, 23, 24) performed SATS and three (15, 22, 25) performed SATV. Compared with SATS, acupuncture showed a significant association with the reduction of the PSQI scores (MD = −3.71, 95%CI = −4.88 to −2.54, *p* < 0.00001, *I* ^2^ = 95%). However, there were no differences between acupuncture and SATV (MD = −2.76, 95%CI = −5.68 to 0.15, *p* = 0.06, *I* ^2^ = 91%) (**Figure 5**).

### 4.3 Acupuncture *vs*. Western medication

**Figure 6:**
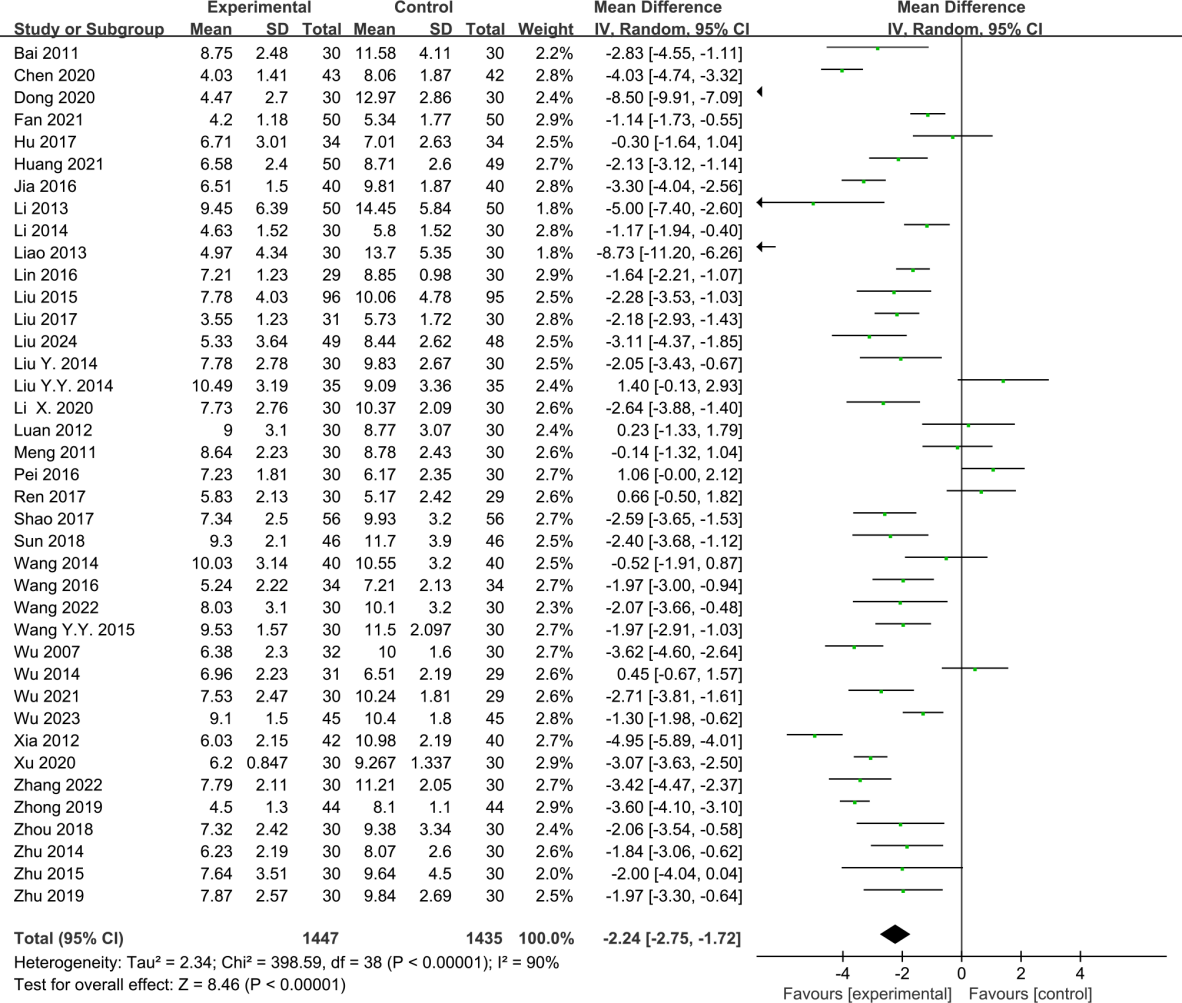
Forest plots of acupuncture *vs*. Western medication for the Pittsburgh Sleep Quality Index (PSQI) after treatment. *WM*, Western medication.

A total of 39 RCTs (26–64) were included. The results of the meta-analysis revealed that acupuncture significantly reduced the PSQI scores more than Western medication (MD = −2.24, 95%CI = −2.75 to −1.72, *p* < 0.00001, *I* ^2^ = 90%) (**Figure 6**). Due to the high heterogeneity, a random effects model was used. In the subgroup analyses, the results for the different acupuncture types (MA or EA) (**Supplementary Figure S5**) and the number of acupoints (≥10 or <10) (**Supplementary Figure S6**) showed that acupuncture had a significant difference compared with Western medication. No significant interaction effects were found in the subgroups (MA *vs*. EA: *Chi* ^2^ = 0.03, *df* = 1, *p* = 0.85; ≥10 *vs*. <10: *Chi* ^2^ = 0.61, *df* = 1, *p* = 0.44). The sensitivity analysis demonstrated the good robustness of the results (**Supplementary Figure S7**). The funnel plots showed apparent asymmetry (**Supplementary Figure S8**), and the *p*-value was 0.002 in the Egger’s test; thus, it was assessed that there may be some publication bias.

### 4.4 Acupuncture dose and the PSQI score

All 56 RCTs were included to investigate potential correlations between efficacy and acupuncture dose (acupuncture frequency, acupuncture session, and acupuncture course).

#### 4.4.1 Acupuncture frequency and the PSQI score

**Figure 7:**
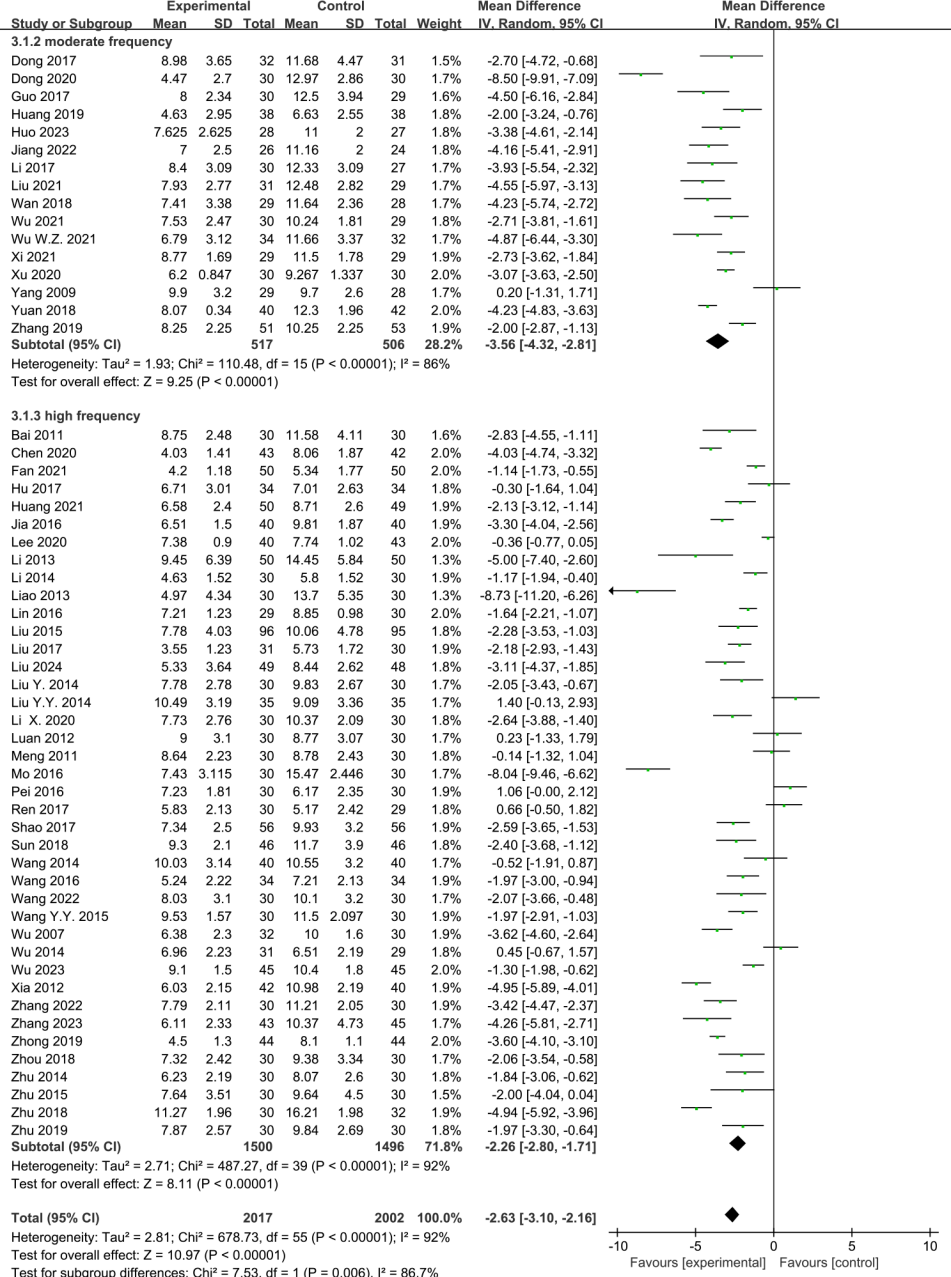
Effects of acupuncture on the Pittsburgh Sleep Quality Index (PSQI) scores based on different frequencies.

There were 16 RCTs (10, 11, 13–22, 24, 55, 57, 59) that reported the effect of moderate-frequency acupuncture and 40 RCTs (9, 12, 23, 25–54, 56, 58, 60–64) that reported the effect of high-frequency acupuncture on PI. The pooled results showed that acupuncture had a significantly greater effectiveness compared with the control group in lowering the PSQI scores, within both the moderate-frequency group (MD = −3.56, 95%CI = −4.32 to −2.81, *p* < 0.00001, *I* ^2^ = 86%) and the high-frequency group (MD = −2.26, 95%CI = −2.80 to −1.71, *p* < 0.00001, *I* ^2^ = 92%) (**Figure 7**).

#### 4.4.2 Acupuncture session and the PSQI score

**Figure 8:**
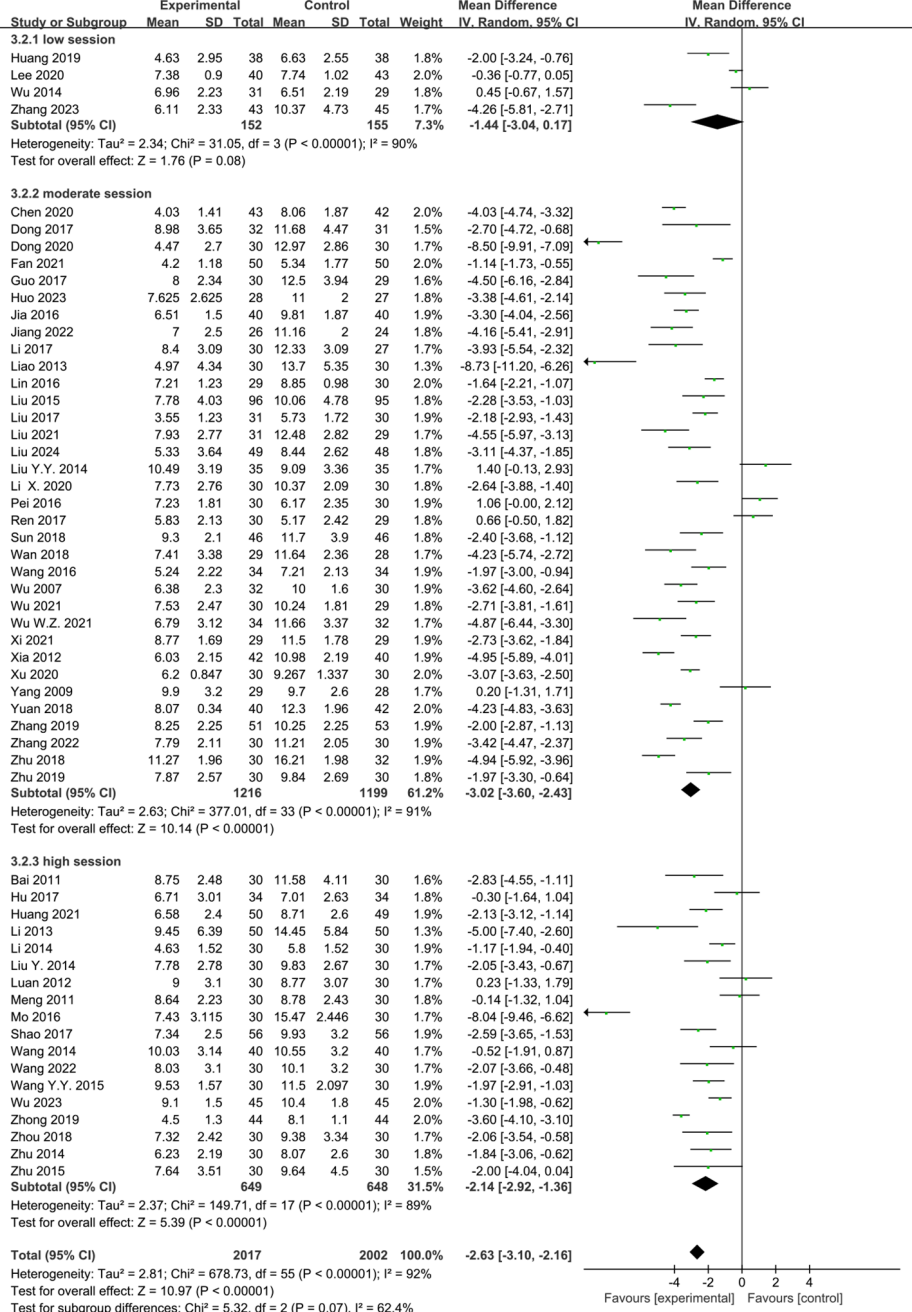
Effects of acupuncture on the Pittsburgh Sleep Quality Index (PSQI) scores based on different sessions.

Of the RCTs, four (14, 23, 25, 37) reported the effect of low-session acupuncture, 34 (10–13, 15–22, 24, 26, 30, 31, 34, 39, 42–45, 47, 49, 51, 53–59, 61, 64) reported the effect of moderate-session acupuncture, and 18 (9, 27–29, 32, 33, 35, 36, 38, 40, 41, 46, 48, 50, 52, 60, 62, 63) reported the effect of high-session acupuncture on PI. The pooled results showed that acupuncture was observed to have a significant difference compared with the control group in the improvement of the PSQI scores in the moderate-session group (MD = −3.02, 95%CI = −3.60 to −2.43, *p* < 0.00001, *I* ^2^ = 91%) and the high-session group (MD = −2.14, 95%CI = −2.92 to −1.36, *p* < 0.00001, *I* ^2^ = 89%). However, there were no differences between the acupuncture and control groups in the low-session group (MD = −1.44, 95%CI = −3.07 to −0.17, *p* = 0.08, *I* ^2^ = 90%) (**Figure 8**).

#### 4.4.3 Acupuncture course and the PSQI score

**Figure 9:**
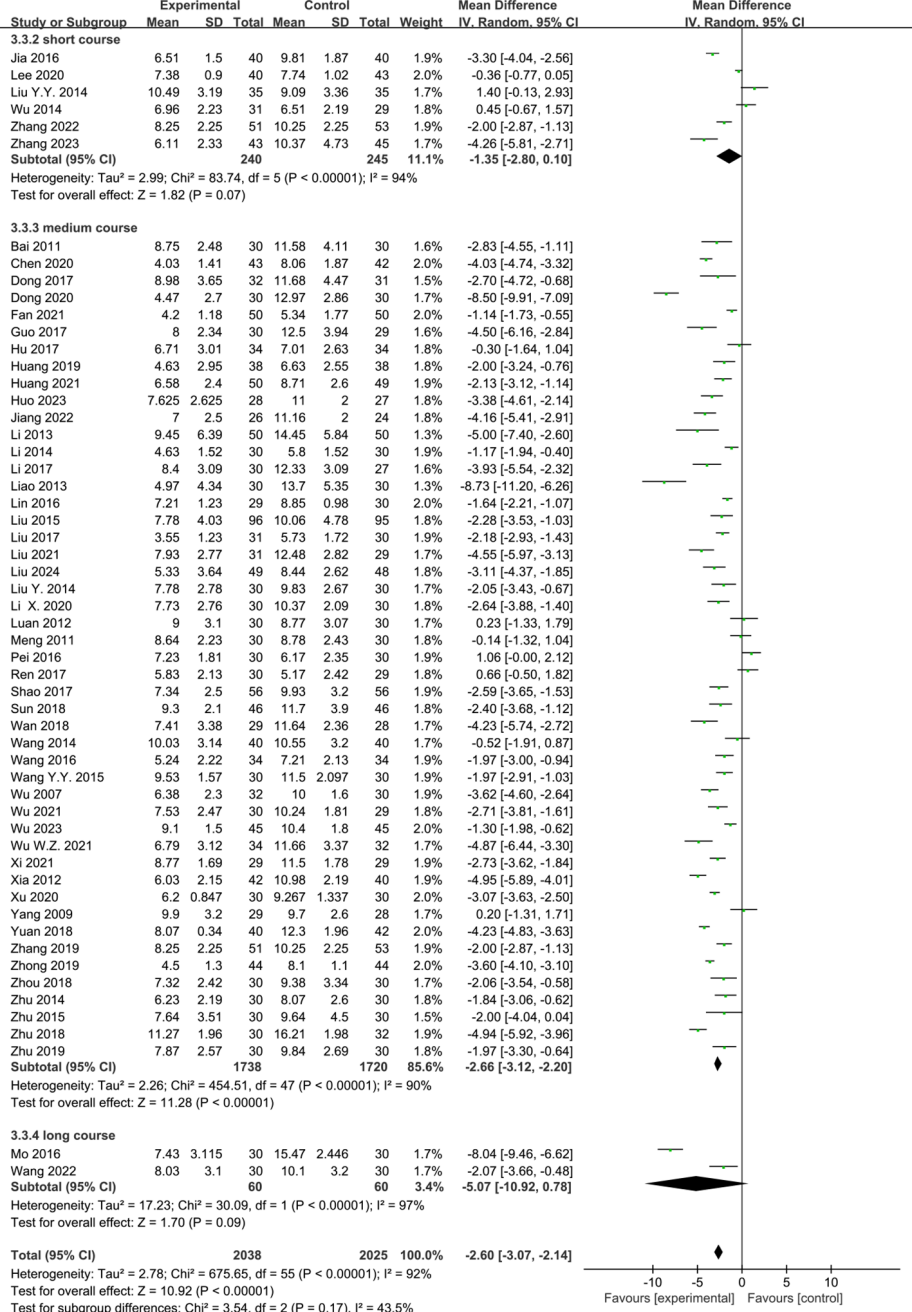
Effects of acupuncture on the Pittsburgh Sleep Quality Index (PSQI) scores based on different courses.

Six RCTs (23, 25, 34, 37, 42, 61) reported the effect of short-course acupuncture on insomnia, 49 RCTs (10–24, 26–33, 35, 36, 38–41, 43–60, 63, 64) the effect of medium-course acupuncture, and two RCTs (9, 62) the effect of long-course acupuncture. The pooled results indicated that acupuncture was more effective than the control group in terms of the PSQI scores in the medium-course group (MD = −2.66, 95%CI = −3.12 to −2.20, *p* < 0.00001, *I* ^2^ = 90%). However, no greater difference than the control group in the short-course group (MD = −1.35 [95% CI −2.80, 0.10], *P* = 0.07, *I^2^* = 94%) and the high-course group was shown (MD = −1.56, 95%CI = −3.38 to 0.26, *p* = 0.09, *I* ^2^ = 93%) (**Figure 9**).

### 4.5 Quality assessment

The available evidence was evaluated using the GRADE tool. The quality of evidence on acupuncture for PI was graded as “moderate, low, or very low.” Details are shown in **Supplementary Table S2**.

## 5 Discussion

### 5.1 Main findings

This systematic review evaluated the impact of acupuncture on the PSQI scores of individuals with PI based on 56 RCTs that included 4,019 participants. The results demonstrated the efficacy of acupuncture in patients with PI, evidenced by the significant reduction in the PSQI scores compared with those for sham acupuncture or Western medication. Despite the significant heterogeneity observed among the studies, the sensitivity analysis revealed good robustness. In the subgroup analyses, the results indicated that there was no significant association between the different acupuncture types and the number of acupoints with regard to efficacy. As is well known, pharmacological treatment highlights accurate drug frequency and the duration of treatment. Similar principles are applied in acupuncture. Therefore, based on the above results, we opted to disregard the differences, assuming equivalent efficacy, and investigated the influence of acupuncture frequency, acupuncture session, and acupuncture course. The findings suggested that at least three sessions per week for 3–4 weeks and a total of at least 12 acupuncture sessions would be the optimal dosage of acupuncture for PI.

In terms of acupuncture frequency, the results showed that moderate frequency (three sessions per week) and high frequency (five to seven sessions per week) were beneficial for the improvement of sleep quality. Therefore, acupuncture might confer better effects when the treatment frequency is at least three sessions per week. For acupuncture session, the effect of low session (≤10 sessions) was not statistically significant. It showed better effects on the reduction of the PSQI scores until moderate session (12–20 sessions) and high session (24–30 sessions) were reached, indicating that the acupuncture treatment needs to reach a certain dose in order to obtain better therapeutic effects. Therefore, an acupuncture dose of ≥12 sessions should be recommended to achieve better clinical therapeutic effects in clinical practice. This finding was consistent with previous research, which showed that 12 or more acupuncture sessions yielded superior results compared with 6–10 sessions (67). With regard to the acupuncture course, the short (≤2 weeks) and long courses (>4 weeks) showed no obvious differences between acupuncture and sham acupuncture or Western medication. Medium course (3–4 weeks) was considered as the optimal course for acupuncture. The analysis indicated that acupuncture demonstrated comparable efficacy to sham acupuncture or Western medication during the initial 1–2 weeks. However, by the third week, the efficacy of acupuncture surpassed that of sham acupuncture or Western medication. For patients with slow or poor response to Western medication, with strong adverse reactions, or those seeking non-pharmacological options, this finding underscored that acupuncture can serve as an alternative treatment option. The results also indicated that treatment duration of at least 3 weeks can serve as a reference point for the onset of action in clinical treatment, aiding clinicians in refining the treatment plans and assessing efficacy. Nevertheless, extending the treatment to more than 4 weeks did not confer additional benefits. This is related to the “after-effect” of acupuncture treatment. In this meta-analysis, acupuncture was shown to still significantly improve sleep quality at the 4-week, 8-week, and 3-month follow-up. Therefore, continuous acupuncture might lead to “fatigue” of the acupuncture points, which greatly reduces its efficacy. Some studies have found that the long course may be associated with better efficacy of acupuncture, which differed from our results (68, 69). However, as the number of studies with long course was small in this meta-analysis, the current evidence is insufficient. Thus, more high-quality RCTs are needed to verify this in the future.

In this meta-analysis, the outcome of sham acupuncture according to the needling points was also investigated to evaluate the specific effects of acupuncture. The results showed that there was a significant difference between acupuncture and SATS. However, no significant difference was observed between acupuncture and SATV in the PSQI scores. Interestingly, the findings were consistent with those of previous meta-analyses of cancer-related pain (70) and chronic nonspecific low back pain (71), which were conducted using the same hypothesis. These results indicated that the clinical outcome of sham acupuncture could differ depending on whether its needling point is the same as that used in the acupuncture group, suggesting that needling at the same acupuncture points may not be regarded as a true placebo control as there is no consideration for acupuncture point specificity. It is possible that sham contact with acupuncture points is a modified form of acupuncture instead of a true placebo. This result examined the point specificity of acupuncture by analyzing the outcome of sham acupuncture according to the needling point. To obtain more credible evidence, it will be necessary in the future to carry out a direct comparative trial of acupuncture and the above different type of sham acupuncture.

### 5.2 Implications for practice and research

The number of studies on acupuncture for insomnia has increased exponentially in recent years; however, the relationship between acupuncture dose and its effect remains unclear. To some extent, this limits the use of acupuncture. For any kind of drug intervention, the relationship between the therapeutic dose and efficacy is critical. Similarly, the relationship between the acupuncture dose and clinical efficacy should be studied and is of great clinical value. This dose–effect meta-analysis suggested that at least three sessions per week for 3–4 weeks and a total of at least 12 acupuncture sessions may be recommended. A prolonged acupuncture course might not increase its efficacy. Therefore, considering the economic burden of patients, when patients with insomnia have agreed to acupuncture treatment for 12 sessions within 3–4 weeks in clinical practice, acupuncture can then be stopped for a period of time to avoid body tolerance. The included studies only used subjective outcome measurements (PSQI score). In order to confirm the conclusion, an objective outcome [polysomnography (PSG) or actigraphy] might be better than a subjective outcome measurement as it is less affected by subjective factors.

### 5.3 Limitations

This meta-analysis has several limitations. Firstly, the number of studies included was limited, particularly for long-course acupuncture, which could have lowered the precision of the results and affected the certainty of the evidence. Secondly, the patients included in the trials had different degrees of sleep disorder, resulting in a possible clinical heterogeneity between studies. Thirdly, the quality of some of the included RCTs was relatively low, which could have influenced the accuracy of the results. Finally, as all but one of the studies were conducted in China, it was considered that there was potential for publication bias as triggered by the cultural background of different regions and countries. Accumulating evidence suggests that the Asia-Pacific region is more inclined toward acupuncture treatment and publishes positive results.

## 6 Conclusions

This systematic review and meta-analysis revealed that acupuncture could play a positive role in the management of insomnia, although the evidence level was very low to moderate. A dose–effect relationship was found between the dose (frequency, session, and course) of acupuncture and the clinical response. Comparison of several different doses will help explore the best treatment options and guide clinical decision making. However, further study is necessary to confirm the dose–effect relationship of acupuncture in PI.

## Data Availability

The original contributions presented in the study are included in the article/**Supplementary Material**. Further inquiries can be directed to the corresponding authors.
