# Supplementary material for: The dose–effect relationship between acupuncture and its effect on primary insomnia: a systematic review and meta-analysis
Source: Front Psychiatry. 2025 Feb 10;16:1501321. doi: 10.3389/fpsyt.2025.1501321 (PMC11847884; doi:10.3389/fpsyt.2025.1501321)
Supplement: Supplementary file 1 [file DataSheet1.docx]

**Supplementary Figures**

FIGURE 1 | Subgroup analysis of different acupuncture methods (acupuncture vs. sham acupuncture) …………………………………………………………………….2

FIGURE 2 | Subgroup analysis of different number of acupoints (acupuncture vs. sham acupuncture) …………………………………………………………………….3

FIGURE 3 | The result of sensitivity analysis (acupuncture vs. sham acupuncture) …4

FIGURE 4 | Funnel plot on publication bias (acupuncture vs. sham acupuncture) …. 5

FIGURE 5 | Subgroup analysis of different acupuncture methods (acupuncture vs. western medication) …………………………………………………………………. 6

FIGURE 6 | Subgroup analysis of different number of acupoints (acupuncture vs. western medication) …………………………………………………………………..7

FIGURE 7 | The result of sensitivity analysis (acupuncture vs. western medication)...8

FIGURE 8 | Funnel plot on publication bias (acupuncture vs. western medication) …9

**FIGURE 1 | Subgroup analysis of different acupuncture methods (acupuncture vs. sham acupuncture)**


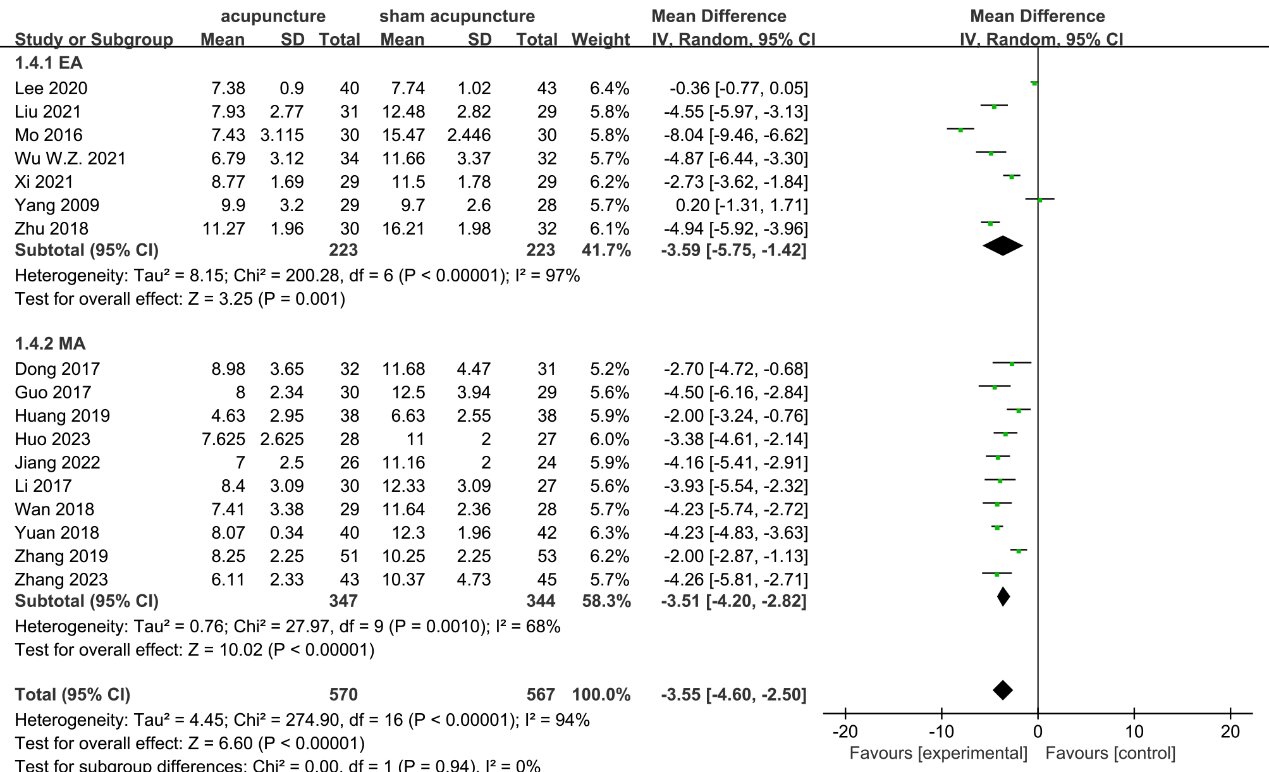


MA, manual acupuncture; EA: electroacupuncture;

**FIGURE 2 | Subgroup analysis of different number of acupoints (acupuncture vs. sham acupuncture)**


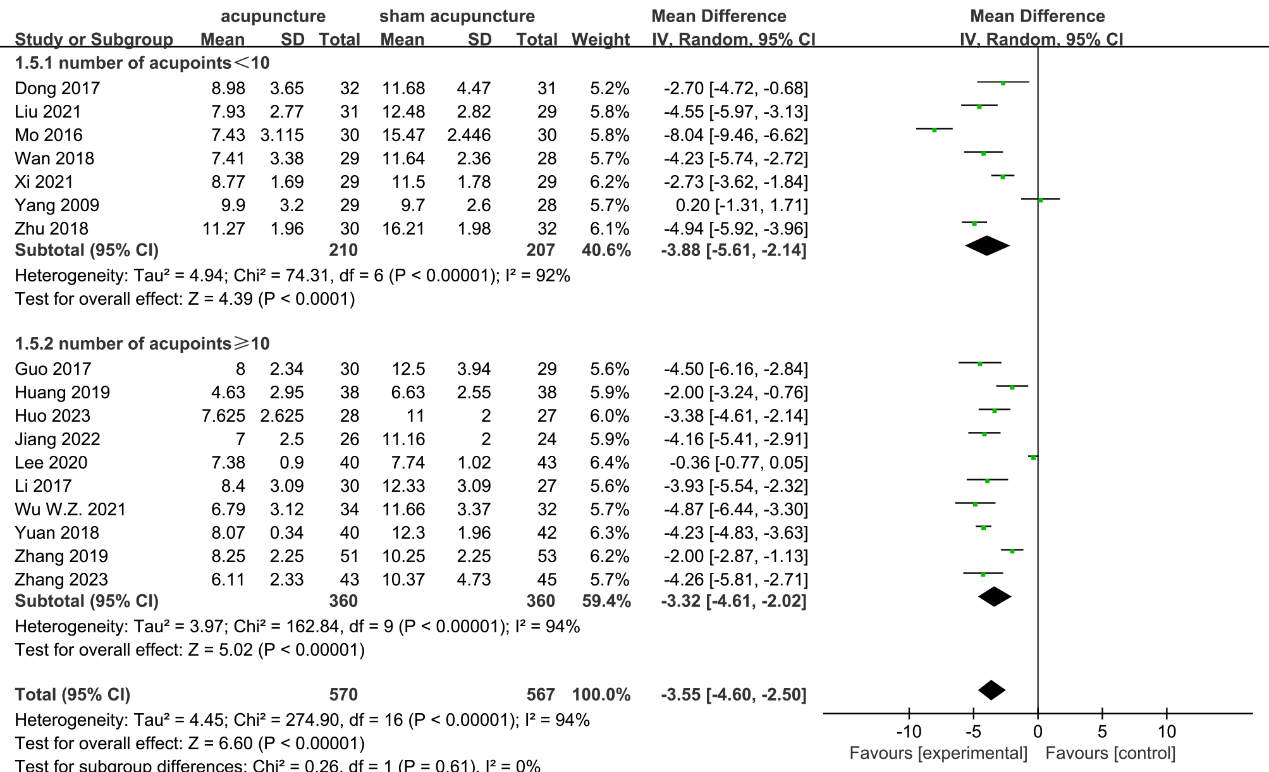


**FIGURE 3 | The result of sensitivity analysis (acupuncture vs. sham acupuncture)**

**FIGURE 4 | Funnel plot on publication bias (acupuncture vs. sham acupuncture)**

**FIGURE 5 | Subgroup analysis of different acupuncture methods (acupuncture vs. western medication)**


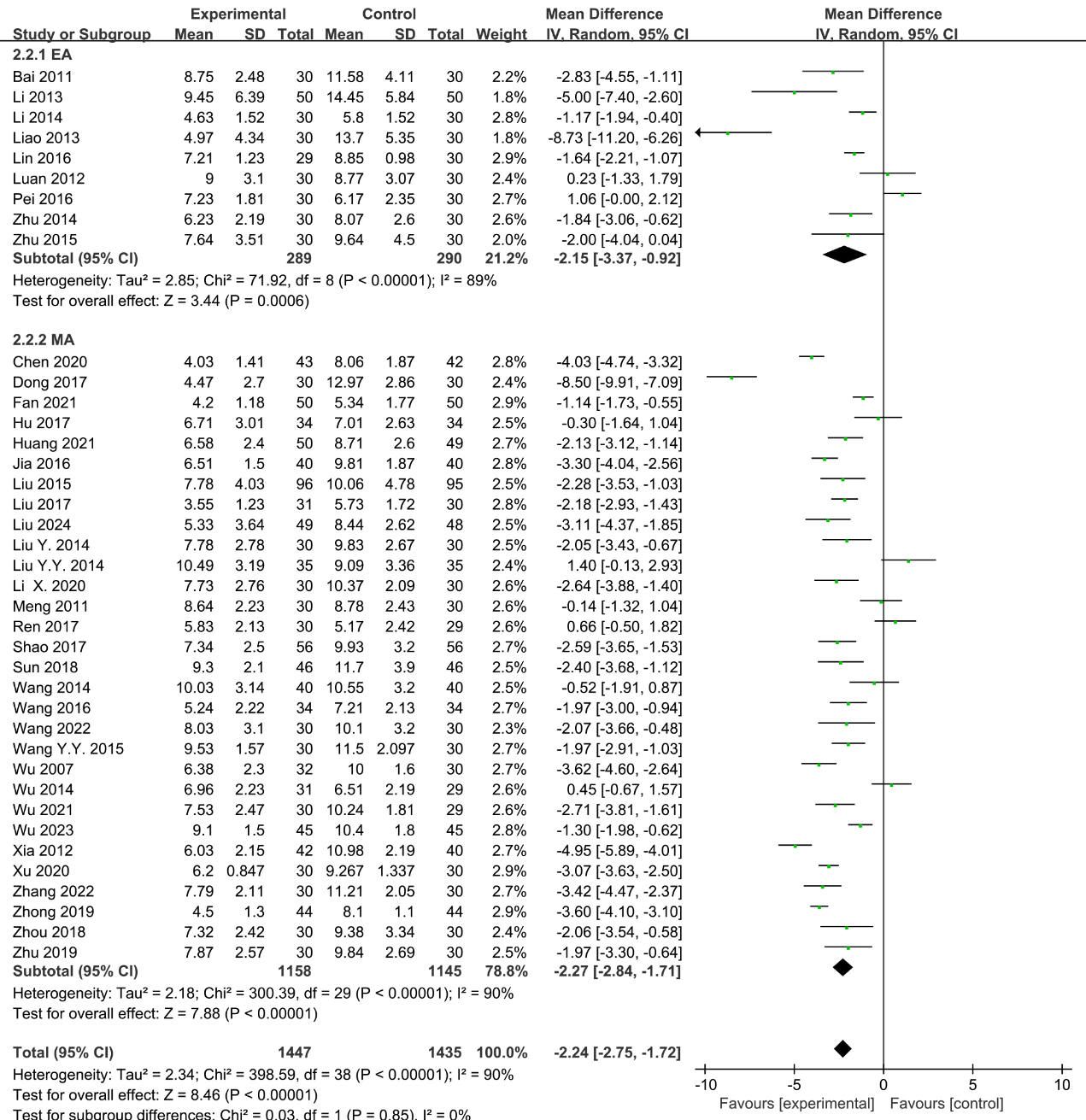


MA, manual acupuncture; EA: electroacupuncture;

**FIGURE 6 | Subgroup analysis of different number of acupoints (acupuncture vs. western medication)**


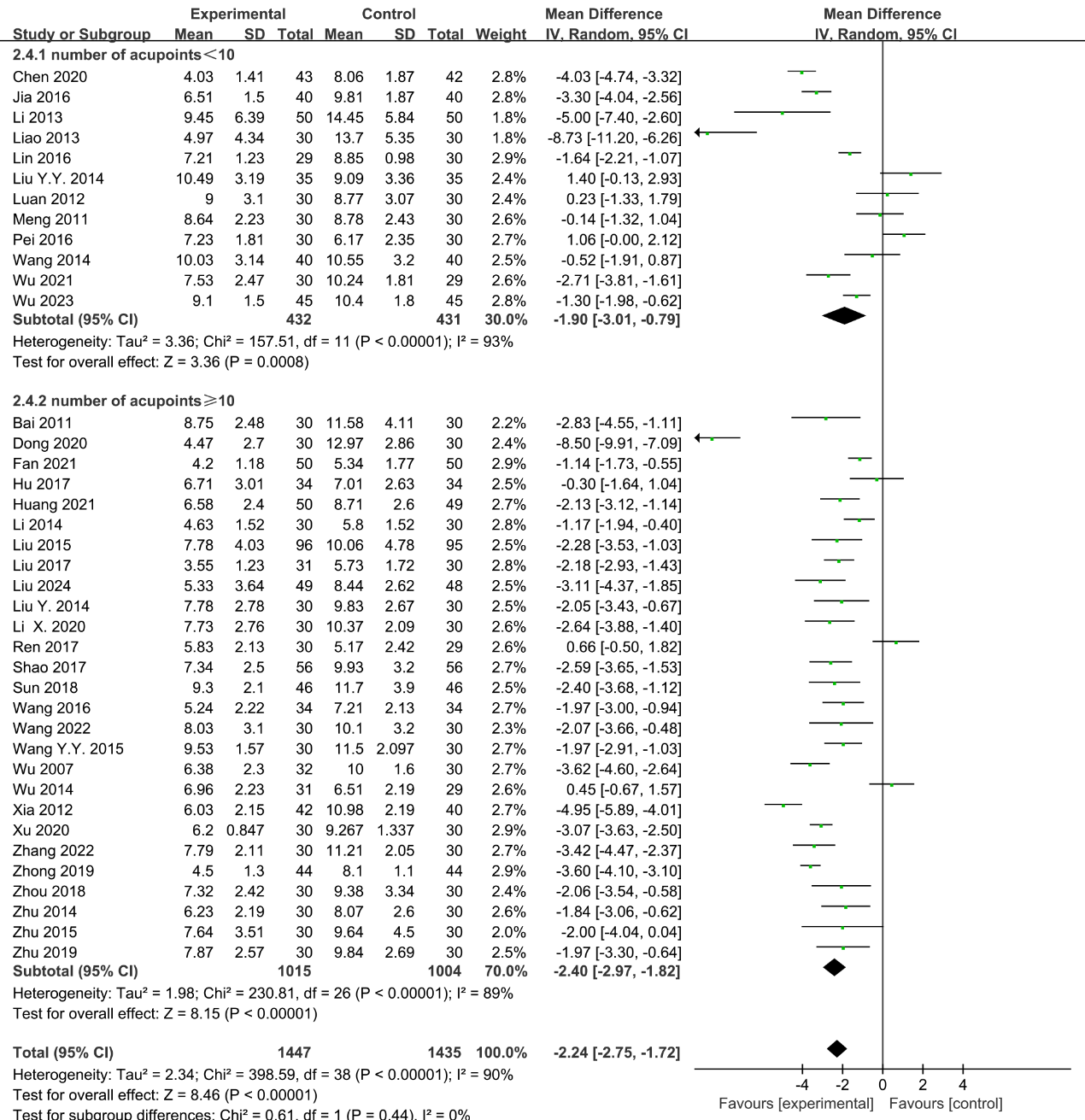


**FIGURE 7 | The result of sensitivity analysis (acupuncture vs. western medication)**

**FIGURE 8 | Funnel plot on publication bias (acupuncture vs. western medication)**
