# Supplementary material for: The dose–effect relationship between acupuncture and its effect on primary insomnia: a systematic review and meta-analysis
Source: Front Psychiatry. 2025 Feb 10;16:1501321. doi: 10.3389/fpsyt.2025.1501321 (PMC11847884; doi:10.3389/fpsyt.2025.1501321)
Supplement: Supplementary file 2 [file DataSheet2.docx]

**Supplementary Tables**

TABLE 1 | Search strategies used for PubMed………………………………………. 2

TABLE 2 | Summary of findings table from GRADE profiler…………………………3

**TABLE 1 | Search strategies used for PubMed**

| No. | Search items |  |
| --- | --- | --- |
| 1 | "Sleep Initiation and Maintenance Disorders"[MeSH Terms] | 18,916 |
| 2 | ("insomnia s"[All Fields] OR "sleep initiation and maintenance disorders"[MeSH Terms] OR ("sleep"[All Fields] AND "initiation"[All Fields] AND "maintenance"[All Fields] AND "disorders"[All Fields]) OR "sleep initiation and maintenance disorders"[All Fields] OR "insomnia"[All Fields] OR "insomnias"[All Fields]) AND (fft[Filter]) | 33,181 |
| 3 | 1 or 2 | 38,396 |
| 4 | "acupuncture"[MeSH Terms] OR "acupuncture therapy"[MeSH Terms] | 31,023 |
| 5 | "acupuncture"[Title/Abstract] OR "Electroacupuncture"[Title/Abstract] OR "manual acupuncture"[Title/Abstract] OR "acupuncture point"[Title/Abstract] OR "acupoint"[Title/Abstract] OR "auricular acupuncture"[Title/Abstract] OR "Electroacupuncture"[Title/Abstract] | 34,077 |
| 6 | 4 or 5 | 40,120 |
| 7 | "randomized controlled trial"[Publication Type] OR "randomized"[Title/Abstract] OR "placebo"[Title/Abstract] | 1,061,549 |
| 8 | 3 and 6 and 7 | 393 |
|  |  |  |

**TABLE 3 | Summary of findings table from GRADE profiler**

**1. acupuncture vs. sham acupuncture**

| **Quality assessment** | | | | | | | **No of patients** | | **Effect** | | **Quality** | **Importance** |  |
| --- | --- | --- | --- | --- | --- | --- | --- | --- | --- | --- | --- | --- | --- |
|  |  |  |  |  |  |  |  |  |  |  |  |  |  |
| **No of studies** | **Design** | **Risk of bias** | **Inconsistency** | **Indirectness** | **Imprecision** | **Other considerations** | **PSQI** | **Control** | **Relative (95% CI)** | **Absolute** |  |  |  |
| **acupuncture VS sham acupuncture (Better indicated by lower values)** | | | | | | | | | | | | |  |
| 17 | randomised trials | no serious risk of bias | serious^1^ | no serious indirectness | no serious imprecision | none | 570 | 567 | - | MD 3.56 lower (4.57 to 2.55 lower) | ⊕⊕⊕O MODERATE |  |  |
| **follow-up - the 4-week follow-up (Better indicated by lower values)** | | | | | | | | | | | | |  |
| 9 | randomised trials | no serious risk of bias | serious^1^ | no serious indirectness | no serious imprecision | none | 295 | 294 | - | MD 4.37 lower (6.21 to 2.53 lower) | ⊕⊕⊕O MODERATE |  |  |
| **follow-up - the 8-week follow-up (Better indicated by lower values)** | | | | | | | | | | | | |  |
| 3 | randomised trials | no serious risk of bias | serious^1^ | no serious indirectness | no serious imprecision | none | 100 | 99 | - | MD 1.74 lower (3.06 to 0.42 lower) | ⊕⊕⊕O MODERATE |  |  |
| **follow-up - the 3-month (Better indicated by lower values)** | | | | | | | | | | | | |  |
| 2 | randomised trials | no serious risk of bias | serious^1^ | no serious indirectness | no serious imprecision | none | 74 | 74 | - | MD 6.23 lower (7.86 to 4.6 lower) | ⊕⊕⊕O MODERATE |  |  |
| **sham acupuncture vs. SATS (Better indicated by lower values)** | | | | | | | | | | | | |  |
| 14 | randomised trials | no serious risk of bias | serious^1^ | no serious indirectness | no serious imprecision | none | 469 | 466 | - | MD 3.71 lower (4.88 to 2.54 lower) | ⊕⊕⊕O MODERATE |  |  |
| **sham acupuncture vs. SATV (Better indicated by lower values)** | | | | | | | | | | | | |  |
| 3 | randomised trials | no serious risk of bias | serious^1^ | no serious indirectness | no serious imprecision | none | 101 | 101 | - | MD 2.76 lower (5.68 lower to 0.15 higher) | ⊕⊕⊕O MODERATE |  |  |

^1^ The high heterogeneity.

**2. acupuncture vs. western medicine**

| **Quality assessment** | | | | | | | **No of patients** | | **Effect** | | **Quality** | **Importance** |  |
| --- | --- | --- | --- | --- | --- | --- | --- | --- | --- | --- | --- | --- | --- |
|  |  |  |  |  |  |  |  |  |  |  |  |  |  |
| **No of studies** | **Design** | **Risk of bias** | **Inconsistency** | **Indirectness** | **Imprecision** | **Other considerations** | **Acupuncture** | **WD** | **Relative (95% CI)** | **Absolute** |  |  |  |
| **acupuncture vs. WM (Better indicated by lower values)** | | | | | | | | | | | | |  |
| 39 | randomised trials | very serious^1,2^ | serious^3^ | no serious indirectness | no serious imprecision | none | 1447 | 1435 | - | MD 2.24 lower (2.75 to 1.72 lower) | ⊕OOO VERY LOW |  |  |

^1^ No detailed description of random methods or allocation of hidden methods.
^2^ Blind method not described in detail.
^3^ The high heterogeneity.

**3. acupuncture dose**

| **Quality assessment** | | | | | | | **No of patients** | | **Effect** | | **Quality** | **Importance** |  |
| --- | --- | --- | --- | --- | --- | --- | --- | --- | --- | --- | --- | --- | --- |
|  |  |  |  |  |  |  |  |  |  |  |  |  |  |
| **No of studies** | **Design** | **Risk of bias** | **Inconsistency** | **Indirectness** | **Imprecision** | **Other considerations** | **Acupuncture dose** | **Control** | **Relative (95% CI)** | **Absolute** |  |  |  |
| **acupuncture frequency - moderate frequency (Better indicated by lower values)** | | | | | | | | | | | | |  |
| 16 | randomised trials | no serious risk of bias^1^ | serious | no serious indirectness | no serious imprecision | none | 517 | 506 | - | MD 3.56 lower (4.32 to 2.81 lower) | ⊕⊕⊕O MODERATE |  |  |
| **acupuncture frequency - high frequency (Better indicated by lower values)** | | | | | | | | | | | | |  |
| 40 | randomised trials | very serious^1,2^ | serious^3^ | no serious indirectness | no serious imprecision | none | 1500 | 1496 | - | MD 2.26 lower (2.80 to 1.71 lower) | ⊕OOO VERY LOW |  |  |
| **acupuncture session - low session (Better indicated by lower values)** | | | | | | | | | | | | |  |
| 4 | randomised trials | serious^1^ | serious^3^ | no serious indirectness | no serious imprecision | none | 152 | 155 | - | MD 1.44 lower (3.04 lower to 0.17 higher) | ⊕⊕OO LOW |  |  |
| **acupuncture session - moderate session (Better indicated by lower values)** | | | | | | | | | | | | |  |
| 34 | randomised trials | serious^1^ | serious^3^ | no serious indirectness | no serious imprecision | none | 1216 | 1199 | - | MD 3.02 lower (3.60 to 2.43 lower) | ⊕⊕OO LOW |  |  |
| **acupuncture session - high session (Better indicated by lower values)** | | | | | | | | | | | | |  |
| 18 | randomised trials | very serious^1,2^ | serious^3^ | no serious indirectness | no serious imprecision | none | 649 | 648 | - | MD 2.14 lower (2.92 to 1.36 lower) | ⊕OOO VERY LOW |  |  |
| **acupuncture course - short course (Better indicated by lower values)** | | | | | | | | | | | | |  |
| 6 | randomised trials | serious^1^ | serious^3^ | no serious indirectness | no serious imprecision | none | 240 | 245 | - | MD 1.35 lower (2.80 lower to 0.10 higher) | ⊕⊕OO LOW |  |  |
| **acupuncture course - medium course (Better indicated by lower values)** | | | | | | | | | | | | |  |
| 49 | randomised trials | very serious^1,2^ | serious^3^ | no serious indirectness | no serious imprecision | none | 1738 | 1720 | - | MD 2.66 lower (3.12 to 2.20 lower) | ⊕OOO VERY LOW |  |  |
| **acupuncture course - long course (Better indicated by lower values)** | | | | | | | | | | | | |  |
| 2 | randomised trials | no serious risk of bias | serious^3^ | no serious indirectness | serious^4^ | none | 60 | 60 | - | MD 5.07 lower (10.92 lower to 0.78 higher) | ⊕⊕OO LOW |  |  |

^1^ Blind method not described in detail.
^2^ No detailed description of random methods or allocation of hidden methods.
^3^ The high heterogeneity.
^4^ The sample size is small.
